# Mineralized Cryogel/Hydrogel Constructs to Recapitulate Early Breast Cancer Bone Metastasis In Vitro

**DOI:** 10.1002/advs.202519798

**Published:** 2026-01-27

**Authors:** Jana Sievers‐Liebschner, Petra B. Welzel, Maximilian Fusenig, Linda Sturm, Dagmar Pette, Wolfgang Wagermaier, Claudia Fischbach, Peter Fratzl, Carsten Werner

**Affiliations:** ^1^ Division Polymer Biomaterials Science, Max Bergmann Center of Biomaterials Dresden Leibniz‐Institut für Polymerforschung Dresden e.V. Dresden Germany; ^2^ Department of Biomaterials Max Planck Institute of Colloids and Interfaces Potsdam Germany; ^3^ Nancy E. and Peter C. Meinig School of Biomedical Engineering Cornell University Ithaca New York USA; ^4^ Kavli Institute at Cornell for Nanoscale Science Cornell University Ithaca New York USA; ^5^ Center for Regenerative Therapies Dresden and Cluster of Excellence Physics of Life Technische Universität Dresden Dresden Germany

**Keywords:** bone colonization, bone‐like mineral, cryogel, in vitro metastasis model, multiphasic hydrogel, tumor microenvironment

## Abstract

Initial stages of bone colonization by breast cancer cells are critical for metastasis, but current in vitro models cannot decipher the microenvironmental cues involved. Therefore, a biphasic hydrogel model system is designed that recapitulates structural, biophysical, and biochemical components of the bone microenvironment to replicate early metastasis events. Breast cancer cells embedded within a glycosaminoglycan‐based nanoporous hydrogel phase are traced as they colonize a directly adjacent macroporous cryogel compartment, precisely and selectively equipped with specific bone‐like biomolecular signals and/or solution‐deposited mineral crystals. Microscopic monitoring of the spatiotemporal cancer cell distributions yields colonization profiles that display the correlated effects of cell invasion, matrix interaction, and proliferation. MDA‐MB‐231 cells, but not MCF‐7 cells, rapidly infiltrate the cryogel compartment at rates depending on the cross‐linking degree of the hydrogel phase. Cryogel functionalization with adhesion‐mediating peptide ligands enhances matrix interactions and survival/proliferation of the MDA‐MB‐231 cells. When combined with cryogel‐released stromal cell‐derived factor 1 (SDF‐1), survival/proliferation are further amplified and additionally MDA‐MB‐231 cell invasion is promoted. The presence of deposited bone‐like mineral strongly impedes these responses and is accompanied by characteristic alterations in distinct cellular gene‐expression programs. The reported methodology may not only provide further mechanistic insights into early bone metastasis, but also facilitate the screening of anti‐metastatic drugs.

## Graphical abstract

A new biphasic in vitro cryogel/hydrogel model of early breast cancer bone metastasis provides precise control over microenvironmental cues and microscopic access to the spatiotemporal cancer cell distribution. Deposited bone‐like mineral is observed to strongly impede the effects of biomolecular signals, as evidenced by both colonization profiles and cellular gene expression.

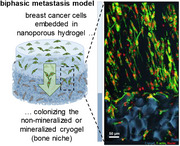

## 1 Introduction

Microenvironmental cues, such as mineral content and properties, adhesiveness, soluble factors, and physicochemical properties, critically control the fate of disseminated cancer cells within bone tissue [1, 2]. While later stages of breast cancer bone metastasis –specifically, the deregulation of bone homeostasis known as the ‘vicious cycle of osteolytic bone metastasis’ – have been extensively studied, the decisive early stages of metastatic bone colonization remain enigmatic and demand more research [2, 3], in particular for the significance and interplay of matrix mineralization and biomolecular cues of the bone tissue [4]. A reduced bone mineral content, often associated with aging or disease, has been linked to an increased risk of bone metastasis [5, 6]. Furthermore, recent mouse studies suggest that bone colonization begins in microenvironmental niches characterized by active osteogenesis [7, 8, 9] and the presence of less mature bone mineral crystals [10, 11, 12]. However, as animal models are limited in microscopic accessibility [13] and do not fully replicate human bone biology, experimental approaches that target these shortcomings are required to explore the initial stages of bone colonization by human breast cancer cells.

In vitro tissue and disease models utilizing synthetic and semi‐synthetic polymer hydrogels can direct cells in a highly controlled and reproducible manner [14, 15, 16]. These models are particularly useful for dissecting the distinct microenvironmental mechanisms involved in the initiation and progression of metastatic bone colonization as demonstrated by in vitro studies on paracrine signaling, the effects of drug treatments, and cancer cell dormancy [17, 18, 19]. In these previous models, cancer cells were seeded either within [17, 20, 21] or on top of hydrogels [19, 22, 23]. These approaches provide limited options for tracing and modulating cell invasion, as they do not realistically simulate how cancer cells move through the diverse structures of the body. Multiphasic in vitro culture systems are composed of multiple, distinct phases designed to more accurately mimic the structural and compositional complexity of natural tissues. They enabled the study of tumor cell extravasation and the impact of tumor stiffness on metastatic potential [24, 25, 26]. Moreover, the physicochemical properties of bone‐like mineral crystals can induce conformational changes in fibronectin and stimulate breast cancer cells to secrete pro‐angiogenic and pro‐inflammatory factors, as demonstrated in studies employing hydroxyapatite‐containing poly(lactide‐co‐glycolide) (PLG) scaffolds [27, 28, 29]. Breast cancer cells cultured on bone‐like intrafibrillar mineralized collagen substrates exhibited increased migratory velocity compared to non‐mineralized substrates, along with reduced adhesion and altered collagen fiber remodeling [30]. Also, in vitro studies using aragonite‐containing biomatrices from the marine invertebrate *Porites lutea* highlighted the relevance of crystalline minerals for breast cancer cell invasiveness and fate [31]. Collectively, these studies clarified the key role of specific microenvironmental parameters to be included in advanced in vitro metastasis models.

**FIGURE 1:**
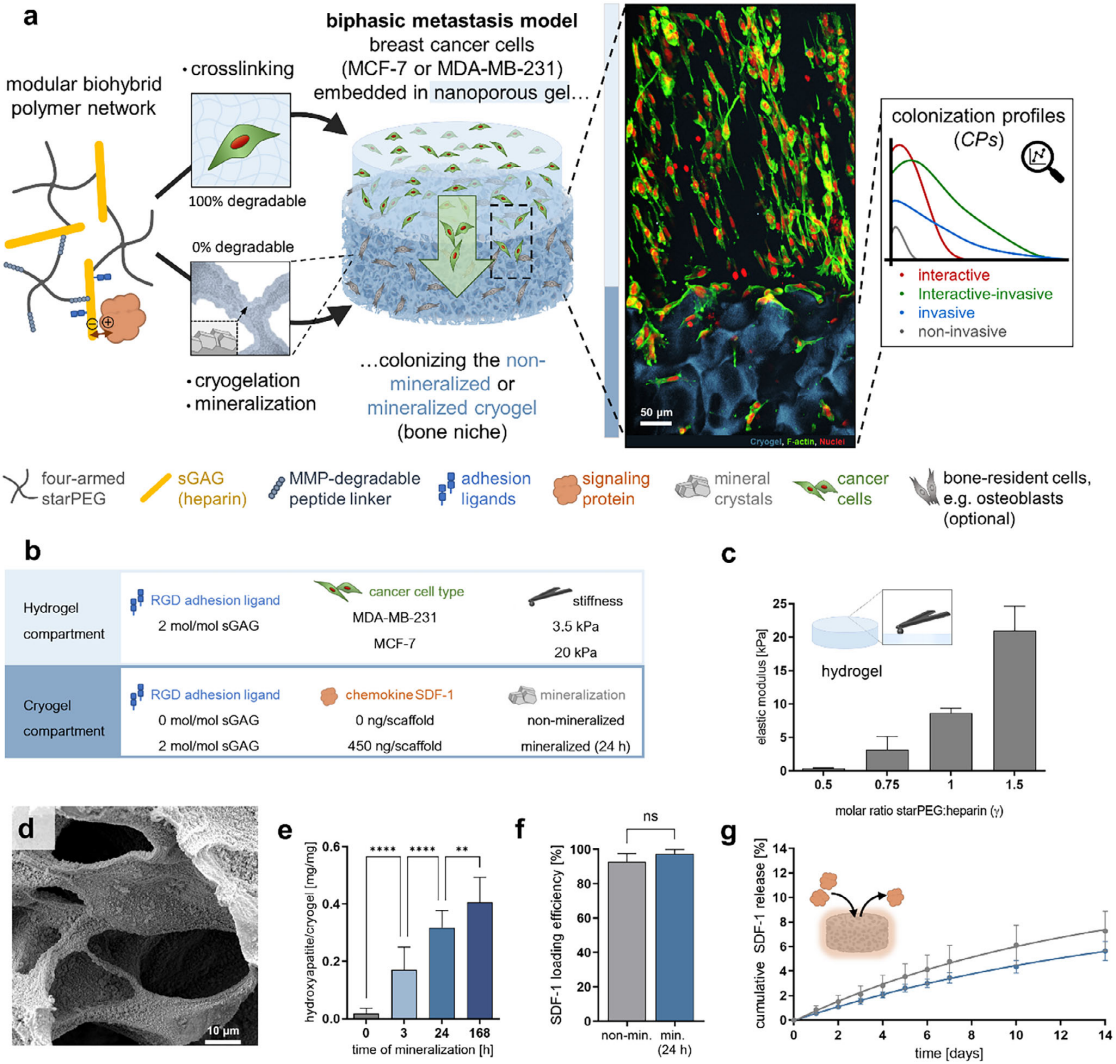
Design and key features of the biphasic in vitro breast cancer bone metastasis model. (a) Composition of the starPEG‐sGAG (mineralized) cryogel/hydrogel constructs, (b) Variable hydrogel and cryogel parameters, (c) Mechanical characteristics of the nanoporous bulk starPEG‐sGAG hydrogel depending on the cross‐linking degree (here: molar ratio of starPEG to heparin, γ), as determined by AFM‐nanoindentation measurements, (d) Exemplary scanning electron microscopy (SEM) image of evenly distributed mineral crystals inside an ex vivo mineralized starPEG‐sGAG cryogel (time of mineralization: 24 h), (e) Mineral content of starPEG‐sGAG cryogels. Data is represented as amount of hydroxyapatite per dry weight of cryogel for various mineralization times, (f) Loading efficiencies of non‐mineralized and mineralized starPEG‐sGAG cryogels for SDF‐1. Per scaffold, 450 ng SDF‐1 were deployed, (g) Cumulative percentage release of SDF‐1 from non‐mineralized (Reproduced with permission [34]. 2021, Elsevier) (gray) and mineralized (blue) starPEG‐sGAG cryogels.

Toward such a more realistic and defined in vitro model of early breast cancer bone colonization that allows microscopic access to cancer cells within the construct, we have designed, fabricated, and applied a novel biphasic cryogel/hydrogel platform. For systematically tuning bone‐like matrix features, a modular hydrogel system composed of four‐armed star‐shaped poly(ethylene glycol) (starPEG) and sulfated glycosaminoglycans (sGAG) − here heparin − was used to create biphasic cell culture constructs made of i) a macroporous cryogel that can be optionally mineralized, and ii) a directly adjacent nanoporous bulk hydrogel phase embedding cancer cells (as shown in Figure 1a). Solution‐based mineralization protocols were adapted for the controlled, gradated deposition of bone‐like mineral on the struts of the macroporous starPEG‐sGAG matrix [32] to dissect the effects and interplay of mineral and biomolecular bone matrix features in cancer cell invasion and homing. The new methodology has proven suitable for the mechanistic in‐depth analysis of critical early bone metastasis events and may become instrumental in the systematic investigation of metastasis‐inhibiting therapies.

## 2 Results and Discussion

### 2.1 StarPEG‐sGAG Cryogel/Hydrogel Constructs as Bone Marrow Endosteal Niche In Vitro Models

During early‐stage bone colonization, disseminated breast cancer cells invade through the bone marrow and its associated matrix before adhering to the mineralized endosteal bone surface [16]. The matrix within bone marrow is soft, primarily consisting of fibrillar and non‐fibrillar collagen, and becomes stiffer closer to the bone surface due to an increased content of fibronectin [33]. To simulate this condition, we developed a biphasic set‐up that consists of breast cancer cells embedded in an enzymatically cleavable starPEG‐sGAG hydrogel matrix (cancer cell reservoir) to invade an adjacent macroporous cryogel compartment. The cross‐linking degree/stiffness of the reservoir hydrogel phase was varied and the cryogel compartment was systematically functionalized by conjugation of adhesion ligand peptides, loading of chemokines and/or deposition of bone‐like mineral crystals (Figure 1).

Both hydrogel compartments are made of the highly sulfated glycosaminoglycan (sGAG) heparin, covalently cross‐linked with starPEG (Figure 1a, left side) [34, 35, 36]. The rational design of the biohybrid starPEG‐sGAG hydrogel platform allows for the independent variation of extracellular matrix (ECM)‐derived biochemical and biophysical cues. In particular, the complexation of bioactive molecules to the sGAG component recapitulates a key functionality of ECM proteoglycans and provides a powerful means to control their spatiotemporal distribution [37]. The incorporation of different cell‐adhesive peptides (e.g., containing the RGD‐motif) and matrix metalloproteinase (MMP)‐cleavable peptide cross‐linkers within the cell‐instructive and ‐responsive matrices has been previously demonstrated to direct vascular endothelial cell morphogenesis as well as tumor cell growth and invasion [38, 39, 40].

The macroporous starPEG‐sGAG hydrogel was prepared by a cryogelation technique (Figure 1a, bottom), which implements the cross‐linking of the hydrogel precursors in aqueous solution via carbodiimide chemistry under sub‐zero temperatures. The formed ice‐crystals act as porogens, creating sponge‐like hydrogel materials (cryogels) with large interconnected pores of 40–300 µm and a porosity of ∼80% [34]. With their unique interconnected porous structure and a porosity equivalent to that of trabecular bone [41], cryogels resemble human bone, which is otherwise challenging to recapitulate [42]. To embed the cancer cells, a nanoporous, degradable starPEG‐sGAG bulk hydrogel was used [35], which was cross‐linked via a cytocompatible Michael‐type addition on top of the cryogel compartment. The cross‐linking degree – which correlates with both the susceptibility for enzymatic cleavage (100% of the cross‐links are degradable) and the mechanical characteristics of the gels – can be modulated by varying the molar starPEG to sGAG ratio, resulting in elastic moduli (E) ranging from 0.5 to 20 kPa (Figure 1c). The struts of the macroporous cryogels are significantly stiffer (E ∼40–∼250 kPa) (Figure S1f), mimicking the stiffness difference that cancer cells encounter when invading from the bone marrow to the bone surface. Additional mechanical properties of the cryogels are presented in the Supporting Information.

To deposit bone‐like mineral crystals on starPEG‐sGAG cryogels, we adapted mineral precipitation protocols using supersaturated buffered calcium phosphate solutions. Equilibrating the cryogels in a CaCl_2_ Tris‐buffered saline solution (pH 7.4) enabled a pre‐association of Ca^2+^ ions with the sGAG resembling the crystal nucleation in the bone matrix in vivo [41]. Subsequently, supersaturation conditions resulted in the formation of stable mineral precipitates on the cryogel struts. Within 24 h, the surface of the cryogel walls was completely covered by the plate‐like mineral crystals (Figure 1d; Figure S1a), which remained stable in the hydrated gels (Figure S1b) and were evenly distributed over the cryogel scaffold (Figure S1e). The stability of the mineral on the cryogels was demonstrated by calcium uptake and release (Figure S1j), in agreement with reports on mineralized biomaterials [30]. As potentially released calcium ions could alter cell behavior, a quantification of calcium ion release or uptake from the surrounding medium was exemplarily performed for mineralized cryogels as described in the Supporting Information. In phosphate buffered saline (PBS) (without calcium ions), a relatively small quantity of calcium ions was found to be released into the surrounding solution, whereas in the case of the calcium (1.8 mm) containing DMEM small amounts of calcium ions were taken up from the medium (Figure S1j). However, in both cases, the detected change in the amount of calcium ions in the surrounding solutions was in the nmol‐range and the release and uptake declined over time, suggesting an overall good stability of the mineral within the cryogels over 14 days of observation. Transmission wide‐angle X‐ray scattering (WAXS) measurements displayed uniform peaks characteristic for hydroxyapatite [43] (Figure S1c) throughout the whole scaffold (Figure S1e). The broader peak width of the mineral in the starPEG‐sGAG cryogels, compared to the sharp peaks of highly crystalline synthetic hydroxyapatite, suggests the formation of less‐crystalline carbonated hydroxyapatite, closely resembling the apatite found in natural bone [29]. High‐resolution synchrotron‐based X‐ray diffraction analysis (Figure S1d, right) showed that the growth rate of mineral crystals followed an exponential curve as reported elsewhere [44]. The crystals reached sizes of up to 43 nm after one week (Figure S1d, left), which is comparable to mineral crystals in natural bones (30–40 nm) [45]. The local elastic modulus of the cryogel struts increased with duration of mineralization (Figure S1f) and the mineralized cryogels were – in contrast to some previously reported mineralized materials [46, 47]– robust and not brittle (Figure S1g–i and Supporting Results).

The cryogels can be additionally equipped with biomolecular signals relevant for the bone niche, e.g., with adhesive glycoproteins – such as fibronectin – via adsorption (Figure S1k) or adhesion‐mediating peptide sequences – such as RGD – via covalent attachment (Figure 1b). In this study, an RGD‐containing peptide sequence was covalently bound to the cryogels (Figure 1b) to ensure comparable amounts of the RGD motif in mineralized and non‐mineralized scaffolds. Bone stromal cells express a variety of growth factors and chemokines, among which the chemokine stromal cell‐derived factor 1 α (SDF‐1) plays a pivotal role in the colonization of breast cancer cells in the bone [48]. As previously shown, the sGAG building blocks of starPEG‐sGAG hydrogels [37] and cryogels [34] allow for effective complexation and customized administration of a wide range of cytokines, chemokines, and growth factors. In the current study, sGAG‐containing cryogels mineralized for 24 h were demonstrated to enable homogeneous uptake of SDF‐1, which was not impeded by the mineral layer, achieving a loading efficiency of approximately 90% (Figure 1f) and exhibiting protein release kinetics comparable to those from non‐mineralized cryogels (Figure 1g). The sGAG component of the hydrogel networks thus mediated a sustained local SDF‐1 administration at concentrations effective for cellular activity (approximately 80–100 ng mL^−1^ SDF‐1 released every 24 h within the macropores) [34, 49].

With the above features, the biphasic constructs enabled in vitro models either with or without mineral − the latter representing conditions at remodeling zones of the bone, while maintaining comparable architecture and biomolecular signals. While the biomolecular functionalization and the degree of cryogel mineralization can be varied widely, we exemplarily selected a subset of very distinct conditions for the preparation of the in vitro models herein investigated further (see Figure 1b) in culture experiments with the more indolent ER+ (estrogen receptor‐positive) breast cancer cell line MCF‐7, which is known to primarily metastasize to bone, and the aggressive, triple negative, mesenchymal‐like MDA‐MB‐231 cell line [50], respectively. Leveraging of the microscopic accessibility of the system, we investigated how the breast cancer cells colonize differently functionalized variants of the biphasic hydrogel constructs. Spatial distributions of cells within the cryogel compartment were quantified as 2D *colonization profiles (CPs)*. These *CPs* are functions representing the local absolute frequency of cells within the cryogel compartment as it varies with the distance from the boundary to the cancer cell reservoir (as depicted schematically in Figure 1a). *CPs* offer a unique quantitative assessment of the correlated dynamics between cell invasion, proliferation, and local matrix interactions, as influenced by microenvironmental cues including physical constraints, adhesiveness, soluble factors, and the degree of mineralization. Additionally, *CPs* reflect the impact of cell‐specific endogenous programs, cell density, and contact time. As such, *CPs* enable the identification of specific colonization types (e.g., invasion‐dominated vs. interaction‐dominated), which may be valuable for future data science approaches.

### 2.2 Recapitulating the Correlated Effects of Cellular Programs and Exogenous Cues in Breast Cancer Colonization

**FIGURE 2:**
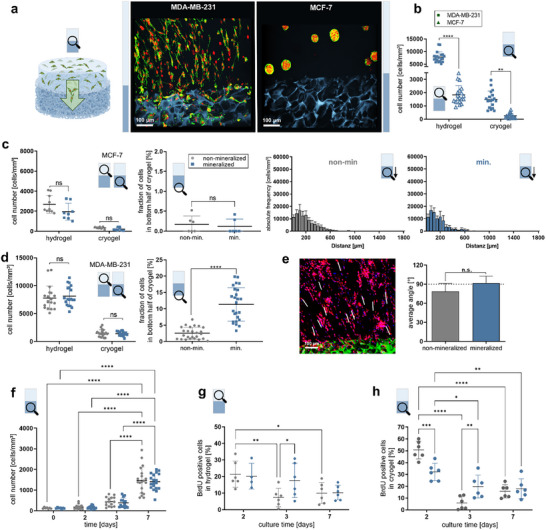
Tumor cell type‐ and mineral‐dependent colonization of biphasic cryogel/hydrogel constructs. (a) Representative cross‐sectional confocal microscopy images (100 µm maximum intensity projections) of the hydrogel (3.5 kPa) to cryogel (non‐mineralized and without biomolecular cues) interface of the biphasic hydrogel system containing MDA‐MB‐231 or MCF‐7 cells after seven days of culture. Red: nuclei, green: F‐actin, blue: cryogel, (b) Quantification of the number of MDA‐MB‐231 or MCF‐7 cells found in the hydrogel or the cryogel compartment after seven days of culture. The number of cells were derived from analysis of confocal microscopy images of nuclei stained samples, (c) Comparison of the culture (7 days) of MCF‐7 cells in the biphasic system using non‐mineralized (gray) or mineralized (blue) cryogels without biomolecular cues. Shown are total cell number in hydrogel and cryogel (left), the quantification of the mineral‐dependent invasion depth of MCF‐7 cells into the cryogels, expressed as percentage of cells found in the bottom half (> 900 µm) of the cryogel relative to the total number of cells present within the cryogel (middle), and the *CPs* showing the distribution of MCF‐7 nuclei after seven days of culture (right), (d) Comparison of the culture (7 days) of MDA‐MBA‐231 cells in the biphasic system using non‐mineralized (gray) or mineralized (blue) cryogels without biomolecular cues. Shown are total cell number in hydrogel and cryogel (left), the quantification of the mineral‐dependent invasion depth of MDA‐MB‐231 cells into the cryogels, expressed as percentage of cells found in the bottom half (> 900 µm) of the cryogel relative to the total number of cells present within the cryogel (right), (e) Analysis of the alignment of MDA‐MB‐231 cells inside the bulk hydrogel compartment. Representative confocal microscopy image (100 µm maximum intensity projection) of the bottom 1000 µm of the hydrogel compartment used for the quantification (green: cryogel, red: F‐actin, blue: nuclei). The white lines exemplarily highlight the orientation of the cells (left). Average orientation of cancer cells (right). The cell orientation was defined with respect to the orientation of the cryogel/hydrogel interface (0°). The F‐actin staining was utilized to visualize the shapes of the cells, (f) Time‐dependent number of MDA‐MB‐231 cells present within the cryogel compartment of the biphasic hydrogel, (g, h) Mineral‐ and time‐dependent changes in proliferative activity of MDA‐MB‐231 cells in the hydrogel (g) and cryogel (h) compartment. Shown is the percentage of BrdU positively stained cells present in the hydrogel or cryogel relative to the total number of cells in each of the compartments.

After seven days of culture within the above‐described constructs, MDA‐MB‐231 cells exhibited an elongated morphology in the hydrogel compartment (Figure 2a,e; Figure S2b), whereas MCF‐7 cells formed spheroid‐like aggregates, which is consistent with previous reports [39]. Quantitative analysis of the cancer cell distribution within the non‐mineralized construct without specific biomolecular cues revealed that a significantly smaller number of MCF‐7 cells invaded into the cryogel compartment compared to MDA‐MB‐231 cells (Figure 2b), aligning with the lower metastatic potential of the MCF‐7 cells. This is also reflected by the *CPs* of the two cell types (Figure 2c, right and 3c, top left) and the very low cell fraction in the bottom half of the cryogel compartment observed for MCF‐7 cells (Figure 2c) in comparison to that found for MDA‐MB‐231 cells (Figure 2d). The cancer cells in the non‐mineralized constructs showed interaction‐dominated *CPs*, with an accumulation of cells at the boundary of the cryogel to the hydrogel compartment. The reduced total number of MCF‐7 cells within the hydrogel compartment (Figure 2b) indicated a lower survival and/or proliferation rate compared to the MDA‐MB‐231 cells.

**FIGURE 3:**
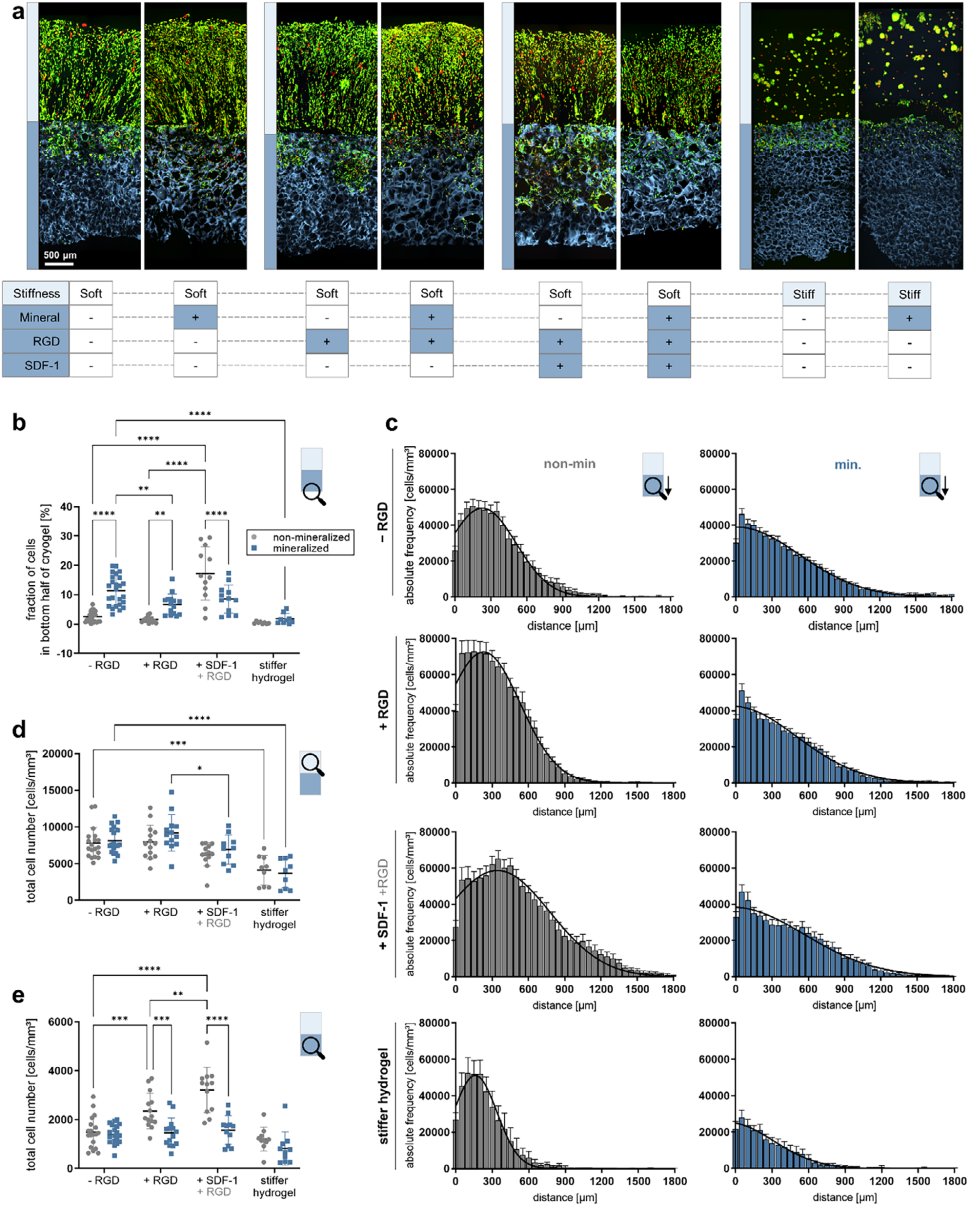
Effects of distinct microenvironmental cues on MDA‐MB‐231 cell invasion. (a) Cross‐sectional confocal microscopy images of MDA‐MB‐231 cells cultured inside biphasic hydrogel constructs for one week. Biomolecular signals (RGD and SDF‐1) were added to the cryogel compartment or cross‐linking degree/stiffness of the hydrogel compartment was modulated (soft: 3.5 kPa, stiff: 20 kPa) in constructs without or with mineral component, (b–e) Quantification of MDA‐MB‐231 cell invasion influenced by the cross‐linking density/stiffness of the nanoporous hydrogel or the additional biofunctionalization of the mineralized and non‐mineralized cryogels with RGD peptides or SDF‐1. Percentage of cells present in the bottom half of non‐mineralized or mineralized cryogels (>900 µm) relative to the total number of cells in the cryogels (b). Colonization profiles representing the distribution of cancer cells within non‐mineralized and mineralized cryogels from top to bottom (average height of cryogel: 1800 µm) (c). Total number of cancer cells in the hydrogel (d) or the cryogel (e) compartment.

To elucidate the influence of bone‐like mineral on the colonization behavior of MCF‐7 and MDA‐MB‐231 cells, biphasic constructs without specific biomolecular cues were investigated in the presence and absence of deposited mineral in the cryogel compartments. While total numbers and *CPs* of MCF‐7 cells remained similarly low after seven days of culture for the mineralized and non‐mineralized constructs (Figure 2c), the *CPs* for the MDA‐MB‐231 cells in the mineralized cryogel compartments clearly differed from the ones in the non‐mineralized constructs (Figure 3c, top) and exhibited a nearly linear trend, indicative of an invasion‐dominated colonization pattern. Notably, a significantly higher number of cells was present in the bottom section of the mineralized cryogels compared to their non‐mineralized counterparts (Figure 2d, right and 3b). This increased migratory activity of MDA‐MB‐231 cells in response to bone‐like minerals is consistent with earlier studies [23, 27, 28, 30]. Nonetheless, the total cell counts in both mineralized and non‐mineralized compartments were similar (Figure 2d), and cell orientation/alignment in the bulk hydrogel compartment remained unaffected by the mineralization status of the cryogel compartment (Figure 2e; Figure S2b).

Analyses of shorter culture periods (0, 2, and 3 days) indicated that the distinct *CPs* for the MDA‐MB‐231 cells in non‐mineralized vs. mineralized cryogels were established early in the culture process (Figure S2c–f). The overall increase of cell number over time in the cryogel and the hydrogel for both systems (Figure 2f; Figure S2d) indicated that colonization of the cryogel is the result of both cell migration and proliferation. While the proliferative activity of MDA‐MB‐231 cells within the hydrogel compartments was similar across all time points examined (2, 3, and 7 days) (Figure 2g), cells on mineralized cryogel substrates initially exhibited less proliferation, likely due to their enhanced migratory behavior (Figure 2h). Conversely, the initially higher proliferation rate of cells in non‐mineralized cryogels decreased significantly after 72 h, whereas the proliferation rate on mineralized cryogels remained steady throughout the evaluated time points. After one week of culture, both conditions showed similar levels of proliferative activity, suggesting that cell–cell interactions and cell‐secreted ECM might mask effects from the underlying cryogel matrices.

Additionally, the cross‐linking degree of the nanoporous bulk hydrogel was found to be critical in regulating both the invasion frequency and proliferation of the cells. As expected, MDA‐MB‐231 cells tended to form spheroid‐like aggregates within the stiffer (20 kPa) bulk reservoir hydrogel (Figure 3a, right), in contrast to the more elongated morphology observed in the softer (3.5 kPa) hydrogel matrix (Figure 3a, left). Moreover, a higher cross‐linking degree of the hydrogel compartment, resulted in a reduced cell density and shallower invasion depth into the cryogel compartment (Figure 3b–e) independent of the presence/absence of mineral. These observations are consistent with those of previous studies [51, 52, 53]. Furthermore, the diminished proliferation in these more confined microenvironments corroborate prior findings regarding the decreased proliferation of MDA‐MB‐231 cells under mechanical constraints [16].

Leveraging the modularity of our model, i.e., its multifunctionality and tunable properties, we delved into the impact of bone‐like biomolecular cues on cancer cell invasion in the absence of bone‐like mineral. Thus, we specifically equipped the non‐mineralized cryogel compartment with RGD‐containing peptide sequences and the chemokine SDF‐1 to assess their effects on the invasion of MDA‐MB‐231 cells. The RGD‐motifs within adhesive glycoproteins of the mineralized bone matrix − such as osteopontin, bone sialoprotein, and fibronectin − facilitate the binding of cells to the mineralized bone matrix [41]. In bone metastasis, these proteins play an important role in mediating the interaction of cancer cells with the bone extracellular matrix. SDF‐1, known for its high expression in the bone microenvironment, is believed to play an important role in the homing and retention of breast cancer cells to bone, particularly as these cells typically express the corresponding receptor CXCR4 [54].

We observed a pronounced interaction‐dominated *CP* for RGD‐functionalized cryogels, as evidenced by a significantly higher total cell count in the cryogel section at the phase boundary to the hydrogel compartment (Figure 3c). Together with the higher total cell number in the cryogel compartment (Figure 3e), this indicates that adhesive interactions promote cell proliferation as previously reported for MDA‐MB‐231 cells [51, 55]. However, RGD functionalization did not lead to increased cell invasion into deeper layers of the cryogel (Figure 3b,c).

The sustained release of the chemokine SDF‐1 from the cryogel, combined with RGD functionalization, led to a similarly interactive but markedly more invasive *CP*. We found that cryogels additionally loaded with 450 ng of SDF‐1 not only further increased proliferation and/or survival (Figure 3e) but also significantly supported the invasion of MDA‐MB‐231 cells into the cryogel compartment (Figure 3b).

Interestingly, the mineralization of the RGD‐functionalized cryogels appeared to obscure the influence of the adhesion ligands. The *CP* of cells within mineralized RGD‐functionalized cryogels closely mirrored the invasion‐dominated *CP* observed in mineralized cryogels lacking RGD (Figure 3c, right). Corroborating our previous findings [30], where we reported that MDA‐MB‐231 cells exhibit reduced adhesion to mineralized collagen substrates irrespective of serum protein presence, our current data further support the notion that the breast cancer cells' response to bone mineral is not primarily governed by the presence of RGD‐motif‐containing adhesion proteins, such as adsorbed fibronectin. SDF‐1's influence on cell infiltration into mineralized, RGD‐functionalized bone‐like environments is less pronounced than for the non‐mineralized counterparts, although the cumulative percentage release of SDF‐1 from non‐mineralized and mineralized starPEG‐sGAG cryogels was found to be not significant different (Figure 1g). Thus, we conclude that SDF‐1 does not significantly interact directly with the mineral, rather the mineral has an influence on the cells and reduces the chemotaxis mediated by SDF‐1.

### 2.3 Gene Expression Analyses and a Theoretical Model Underpin the Observed Colonization Patterns

To further investigate the causes of the above‐reported effects, we analyzed the gene expression in MDA‐MB‐231 cells grown on cryogels with/without mineral for three days − the early time point during culture at which distinct *CPs* were already established − and adapted a theoretical chemotaxis model for the cryogel infiltration process.

**FIGURE 4:**
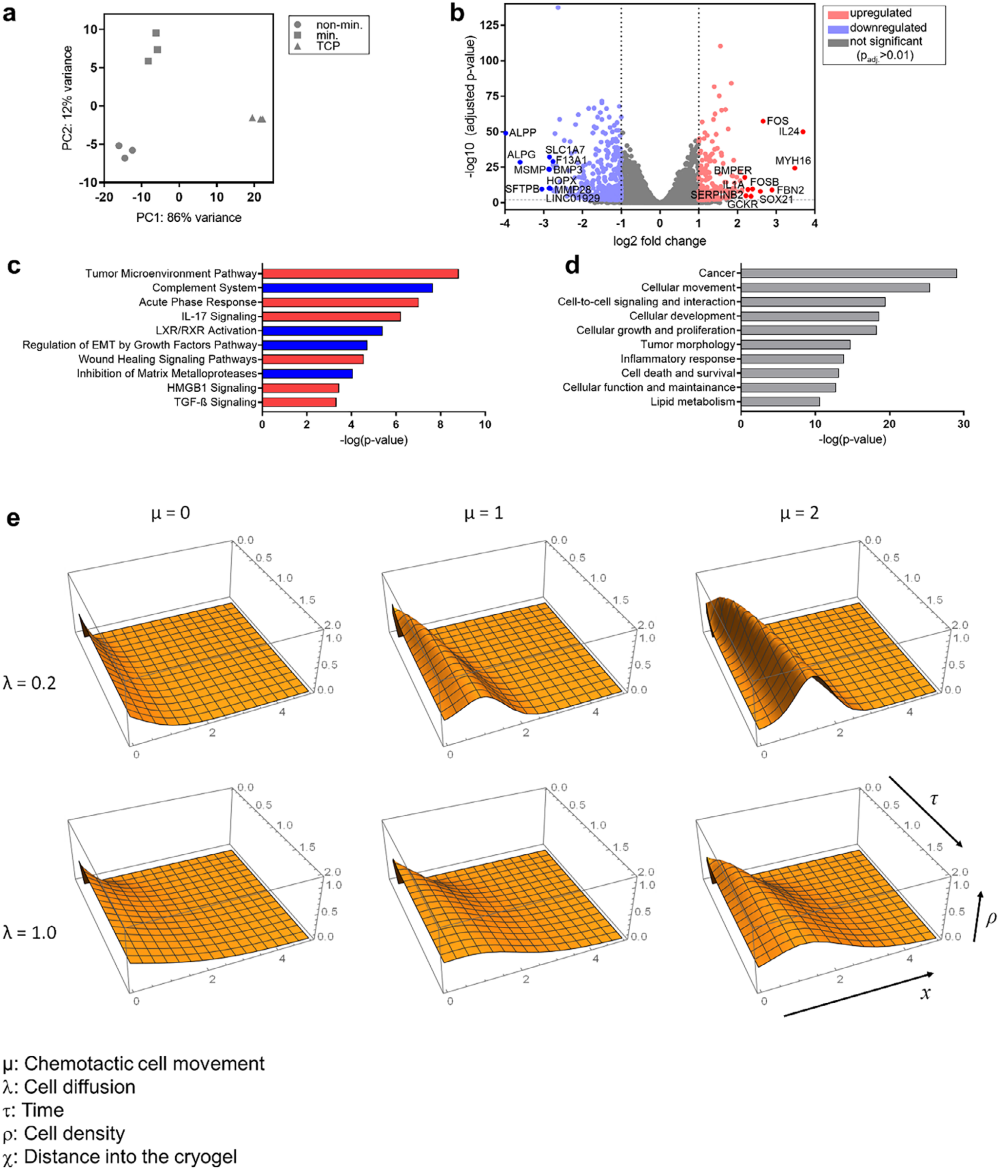
Underpinning the colonization data by gene expression analyses and a theoretical chemotaxis model. (a–d) RNA‐sequencing analysis of MDA‐MB‐231 cells grown for three days in non‐mineralized or mineralized cryogels. As a 2D control, cells were additionally cultured on tissue culture plastic (TCP). Principal component analysis plot visualizing sample‐to‐sample distances of the top 500 most diverse genes (a). Volcano plot of the top 50 (10 labeled genes) most differentially expressed genes in mineralized compared to non‐mineralized cryogel samples with red/blue dots representing differentially expressed genes (DEGs) based on a false discovery rate of 1% and a log2‐fold change of |1| (b). Top 10 up‐ or down‐regulated signaling pathways (c) and diseases and functions (d) associated with the differential gene expression patterns derived from QIAGEN's Ingenuity Pathway Analysis tool (significance threshold of adjusted p‐value ≤ 0.01), (e) Simulation of cell movement based on Keller–Segel‐model [72]. Solutions of Equation 1 for several values of λ and µ, where ρ, τ, x, λ, µ are non‐dimensional parameters proportional to cell density, time, distance into the cryogel, cell diffusion, and chemotactic cell movement in the cryogel compartment, respectively.

Whole transcriptome RNA sequencing (RNA‐seq) of MDA‐MB‐231 cells cultured for three days in mineralized or non‐mineralized cryogels, as well as on tissue culture plastic (TCP), coupled with principal component analysis (PCA) of the RNA‐seq data, revealed that the most substantial differences were observed between cells grown on the 2D TCP control and those in non‐mineralized 3D cryogel scaffolds (Figure 4a, PC1: 86%), constituting 12 141 differently expressed genes (DEGs). Among the top 50 genes upregulated in 3D cryogel scaffolds (Figure S3b,c) were several associated with mitochondrial oxidative phosphorylation (e.g., MT‐CO1, MT‐ND6, MT‐ATP6), mitochondrial protein synthesis (MT‐RNR2), and cytoskeletal organization, adhesion, and migration (AHNAK, CAPN2, ITGA3, ITGA10, ITGB3, PLEC, LMNA, VMN, SQSTM1). In parallel, 3D culture induced the expression of epithelial‐mesenchymal transition (EMT) regulators (AXL), mucins (MUC1, MUC5B, MUC5AC), YWHAZ, and CSF1 (modulating niche cells in the bone). Upregulation of secreted mediators with instructive or pro‐metastatic roles (APOE, SERPINF1, TFF3, LRP1), together with elevated modulation of medium‐borne insulin‐like growth factors (IGFs) by breast cancer‐derived IGF‐binding proteins (e.g., IGFBP4), collectively indicates enhanced metastatic fitness and colonization potential of breast cancer cells in 3D culture, consistent with previous observations by others [56, 57]. This phenotype was further supported by upregulation of proteases (CAPN2, MMP‐13), MMP inducers (BSG), and autophagy regulators (SQSTM1), suggesting a coordinated invasive‐autophagic program in MDA‐MB‐231 cells within 3D cryogel scaffolds.

In addition, the mineral deposition onto cryogel struts significantly influenced gene expression in MDA‐MB‐231 cells (Figure 4a–d; Figure S3). In total, 4255 DEGs were identified between mineralized and non‐mineralized cryogel scaffolds, which were analyzed via QIAGEN's Ingenuity Pathway Analysis (IPA) tool for disease and function categorization, signaling pathway identification, and mechanistic linking.

Categorization of DEGs according to disease and function (Figure 4d; Figure S4b) revealed that the presence of bone‑like mineral in the culture environment promoted a more invasive tumor phenotype. This phenotype correlated with enhanced expression of genes related to inflammation and extracellular matrix (ECM) remodeling, as well as reduced expression of genes involved in cell adhesion and cell–cell contact formation. The observed increase in invasiveness in biphasic scaffold experiments supports this transcriptomic trend. Altered inflammatory signaling likely contributed to the shift toward a more aggressive behavior, consistent with the induction of tumor‑microenvironmental and wound‑healing responses (via functions: proliferation, expansion, and infiltration of cells). Furthermore, reduced expression of integrin‑related genes, together with changes in adhesion dynamics, indicates that mineralization modulates cell–matrix interactions and may alter chemotactic signaling mechanisms.

The categorization of DEGs into broader functions and disease was underpinned by the identification of relevant up‐ and down‐regulated signaling pathways in breast cancer metastasis to bone (Figure S4a). The complement system emerged as the most significantly inhibited pathway. Although the complement cascade contributes to tumor cell proliferation and cancer progression, the specific activators involved within the tumor niche remain incompletely understood [63]. Further, the regulation of EMT by growth factors pathway was downregulated indicating a trend away from soluble factor‐mediated induction of EMT, that is in this particular pathway driven via FGF/FGFR, FOS, MAPK, PDGF, and TNFS members, amongst others. Further, retinoid‐regulated gene programs were downregulated, implying minor involvement of cholesterol and lipid metabolism in breast cancer cells upon invasion, homing, and subsequent migration in mineralized cryogel scaffolds (LXR/RXR activation pathway). In contrast, pathways associated with the tumor microenvironment and inflammation, such as the acute phase response and IL‑17 signaling, were among the most prominently activated in cells cultured in mineralized scaffolds. Many of these pathways converge on the regulation of ECM turnover and cytokine signaling, implicating MMPs and, in particular, interleukins as central mediators of the mineral‑induced cell response.

Among the top 25 DEGs, several factors were directly linked to breast cancer bone metastasis. In more detail, genes encoding inflammatory and signaling factors such as CSF3, IL‐1α, IL‐1β, IL‐6R, and IL‐11, as well as ECM‑degrading enzymes MMP‐1, MMP‐3, MMP‐28, and ADAMTS1, were notably implicated (Figure 4c; Figure S3f). CSF3 has been identified as a key driver of breast cancer cell invasiveness [58] that supports the formation of a tumor‑promoting microenvironment by modulation of bone niche‐residing cells [59]. Interleukins, particularly IL‑1α and IL‑1β, play critical roles in early metastatic progression to bone [60]. IL‑1β stimulates the secretion of secondary inflammatory mediators, including IL‑6 and IL‑8, which coordinate the interaction between cancer stem cell regulation and the surrounding stroma [64–66]. Consistent with this, increased IL‑8 secretion has previously been reported in breast cancer cells cultured in mineralized matrices [28], and IL‑1β has been shown to enhance the formation of cancer stem cell colonies in bone‑like environments [67].

Further, our transcriptomic analysis identified alkaline phosphatase (ALPP and ALPG) to be most significantly downregulated in MDA‐MB‐231 upon culture in mineralized cryogels. As lower expression of alkaline phosphatase is associated with a more malignant phenotype in breast cancer cells [62], our data suggests that mineralized cryogel scaffolds instruct a more aggressive, and invasive, phenotype in MDA‐MB‐231 cells via stark downregulation of ALPP (log2‐fold change: −3.98) and ALPG (log2‐fold change: −3.61) to about 6% and 8% expression of non‐mineralized control scaffolds, respectively [62].

Additional changes included upregulation of stemness‐asociated genes CD274 [68] and ALDH1A3 [69], adjusted gene expression programs orchestrated by FOS/FOSB (modulating osteoclastogenesis and metastasis [61]), HOXB9, MKX, SOX21, concomitant reduction of tumor suppressors (HOPX, SERPINF1, TFF3), and an active inflammatory/remodeling program (IL‐1α, IL‐24, CSF3, MMP‐1, ADAMTS1) with increased expression of anti‐apoptotic factors (BCL2A1, HSPA6, SERPINB2), altogether suggest a shift toward a more stem cell‑like, therapy‐resistant phenotype (Figures S4a and S3e,f). Our findings are congruent with previous in vitro and in vivo studies demonstrating that bone‑derived cues − such as here studied mineralization − can promote breast cancer metastasis and stemness [70, 71]. Lower expression of alkaline phosphatases and integrin‑coding genes (ITGB3, ITGB2, ITGA10) and the SDF‐1 receptor CXCR4 suggest lowered adhesion and dampened responsiveness to niche‐derived SDF‑1 in the mineralized bone niche, indicating an invasory behavior of breast cancer cells upon homing into mineralized microniches (macropores) inside 3D cryogel scaffolds (Figures S3c,e and S4). As the transcriptome analysis was performed three days after culture initiation, our data suggests that cultured breast cancer cells have invaded and fully homed to mineralized microniches (macropores) inside scaffolds, and thus initiated adaptive gene expression programs at the time of analysis (that is, three days post seeding). Together, these observations imply that matrix mineralization promotes a transcriptional reprogramming of MDA‑MB‑231 cells following microniche colonization inside 3D cryogel scaffolds, steering them toward a more invasive and inflammation‑associated phenotype that integrates stemness traits and enhanced survival potential.

Next, we employed a simple Keller–Segel (KS) model of chemotaxis [72], to better understand how breast cancer cell invasion in the cryogel compartment is modulated by the interplay of a cell diffusion‐like or a chemokine‐driven movement.

Based on our own previous work [34], we can assume that the concentration of chemokines in the macroporous system of cryogels is uniform. The KS model posits that the cryogel compartment serves as a source of the chemokine, which diffuses into the hydrogel that initially does not contain this chemokine. The established chemokine concentration gradient (Figure S5) attracts cancer cells initially present only in the hydrogel compartment. The cryogel compartment is assumed to be initially devoid of cancer cells, and as cells migrate across the boundary from the hydrogel, they move in a diffusion‐like manner through the macroporous system of the cryogel compartment. Thus, the KS model considers the superposition of two processes: the diffusion‐like movement of the cancer cells in a cell concentration gradient and the cancer cell movement in a chemokine gradient.

The model's specifics are provided in the Supporting Information, finally resulting in Equation 1:

(1) $$\frac{\partial \rho }{\partial \tau }=-\frac{\partial J}{\partial x},\mathrm{with}\;J=-\lambda \frac{\partial \rho }{\partial x}+\frac{\mu \rho }{\sqrt{\tau }}e^{-x^{2}/\tau }$$

where *ρ*, *τ*, *x* are non‐dimensional parameters proportional to cell density, time, and distance into the cryogel, respectively.

The first term is the diffusion‐like movement of the cells and the second term is the cell movement in a chemokine gradient (that depends on time). The (dimensionless) constants λ and μ are just abbreviations describing diffusion and chemotaxis, respectively. We considered different scenarios in our model: chemotactic sensitivity and diffusivity of the cells in the cryogel compartment were systematically varied. In Figure 4e, the solution for ρ(τ, *x*) for several values of λ and μ were plotted. In the case without chemotaxis (μ  =  0), i.e. pure diffusion‐like movement of cells, starting from an initial cell concentration of zero within the cryogel compartment, a characteristic diffusion gradient emerges over time. Faster diffusion (as described by a larger value of λ) leads to a deeper penetration into the cryogel at any given time. With increasing chemotaxis (as described by a larger value of μ), cancer cells tend to accumulate right below the boundary between hydrogel and cryogel − akin to a “traffic jam” − (peak in ρ at small *x* in Figure 4e). This effect is the more pronounced, the lower the diffusivity of the cells. The model illustrates very clearly which parameters can be varied to control the invasion behavior of the cancer cells.

Although we did not directly fit the experimental data (*CP*s) shown in Figure 3c to the KS model, a qualitative assessment suggests that the presence of mineral in the cryogel compartment correlates with increased cell diffusivity and a decreased chemotactic sensitivity, thereby circumventing the “traffic jam” effect observed in non‐mineralized cryogels (maximum in the *CP*s) under conditions of lower diffusivity (e.g., due to higher adhesion to cryogel‐coupled RGD/more cell–matrix interactions) and higher chemotactic sensitivity to cryogel‐released SDF‐1.

## 3 Conclusion and Outlook

The reported biphasic in vitro model of early breast cancer bone metastasis provides microscopic access and precise and independent control over microenvironmental cues and is therefore ideally suited to dissect the response of different breast cancer cells to adhesion receptor ligands, chemokines, and bone‐like mineral. A significantly smaller number of MCF‐7 cells invaded into the cryogel compartment compared to MDA‐MB‐231 cells, consistent with the lower metastatic potential typically associated with MCF‐7 cells. Direct contact with mineral triggered a distinctly invasive phenotype in metastatic MDA‐MB‐231 cells, as evidenced by significant changes in metastasis‐associated gene expression peograms. Conditions characteristic of high bone turnover − such as reduced mineralization and increased RGD‐mediated adhesion (e.g., to fibronectin, osteopontin, bone sialoprotein [73]) − were found to stimulate MDA‐MB‐231 cell survival/proliferation and additional SDF‐1 signals MDA‐MB‐231 cell survival/proliferation and invasion, potentially facilitating metastatic expansion. Data obtained reinforce the notion that bone matrix mineralization is pivotal in modulating the response of MDA‐MB‐231 cells to biomolecular signals that guide metastatic bone colonization.

The reported methodology is concluded to advance the mechanistic examination of early breast cancer bone metastasis. To unravel the impact of mineralization on relevant signaling pathways such as SDF‐1/CXCR4 on the molecular level, receptor localization could be investigated via immunofluorescence co‐staining in future studies.

Moreover, our approach holds promise for the systematic evaluation of antimetastatic therapies, including personalized analyses. The inclusion of mineral within starPEG‐sGAG cryogels is expected to enhance the relevance of drug assessments, particularly since drug–mineral interactions can influence pharmacological efficacy [74]. To this end, we aim at downsizing the constructs to fit in multiwell plates for integration into robotic liquid handling systems and implementing high‐throughput combinatorial screenings [75] utilizing the model´s sophisticated modularity. A prior study demonstrating the ready‐to‐use capability of pre‐biofunctionalized macroporous starPEG‐sGAG cryogels [34] supports the feasibility of this approach.

Additional facets of this perspective include the targeted collection of invading cell subpopulations from cryogel compartments for detailed analysis, e.g., of integrin expression on the surface of the MCF‐7 and MDA‐MB‐231 cells via flow cytometry or western blot. Furthermore, pre‐seeding the cryogel compartments with bone‐resident cells (Figure 1a), e.g., osteoblasts, osteoclasts, and bone marrow mesenchymal stem cells, or adding components of the immune microenvironment or the vascular system could enhance the model's predictive accuracy.

## 4 Experimental Section

### 4.1 Preparation of MMP‐Degradable Nanoporous starPEG‐sGAG Bulk Hydrogels

Bulk hydrogels were prepared similar to described [35]. Briefly, heparin (Mw: 14 000 g mol^−1^, Merck Millipore, Germany) was conjugated with an average of six maleimide groups (N‐(2‐aminoethyl)maleimide trifluoroacetate salt, Sigma–Aldrich Merck KGaA, Germany) per heparin molecule. Each arm of the starPEG polymer (4‐arm PEG‐maleimide, Mw: 10 000 g mol^−1^, JenKem Technologies) was covalently linked to an in‐house synthesized MMP‐cleavable peptide (Ac‐CGGPQGIWGQGGCG) via one cysteine residue, leaving the second cysteine available for subsequent reaction with the maleimide‐conjugated heparin. For hydrogel formation, the starPEG‐peptide conjugate and the heparin‐maleimide were separately dissolved using PBS. Subsequently, in‐house synthesized cysteine containing RGD‐peptides (GCWGGRGDSP‐Ac, Mw: 990 g mol^−1^) and the cells were directly added to pre‐dissolved heparin‐maleimide. For the hydrogel formation, equal volumes of precursor solutions were mixed and quickly added onto hydrophobic glass coverslips for polymerization. The final hydrogel solution contained a heparin concentration of 1.5 mmol L^−1^ and the molar ratio of starPEG‐peptide conjugate to heparin was varied between 0.5 and 1.5. Glass coverslips were made hydrophobic via treatment with Sigmacote (Sigma–Aldrich Merck KGaA, Germany). For assembly of the biphasic hydrogel constructs, hydrogels with a molar ratio of starPEG‐peptide conjugate to heparin of 0.75 and 1.5, corresponding to elastic moduli of 3.5 and 20 kPa, respectively, were used.

### 4.2 Preparation of Macroporous starPEG‐sGAG Cryogels

Cryogels with a molar ratio of starPEG to heparin of two and a heparin concentration of 3.92 mmol l^−1^ were produced with or without fluorescence label as previously described [34]. Briefly, a hydrogel precursor solution was prepared using four‐armed star‐shaped amino‐end functionalized poly(ethylene glycol) (starPEG‐NH_2_) and heparin, employing the zero‐length cross‐linking agent 1‐ethyl‐3‐(3‐dimethylaminopropyl)‐carbodiimide (EDC, Iris Biotech GmbH, Germany) in combination with N‐hydroxysulfosuccinimide (sulfo‐NHS, Sigma–Aldrich Merck KGaA, Germany). For the formation of fluorescently labeled starPEG‐heparin cryogels, 0.1% (wt w^−1^) of heparin in the hydrogel reaction mixture was replaced with heparin that was prior conjugated with Atto dyes (ATTO‐TEC GmbH, Germany). For all experiments, cryogel discs with a 1 mm height and a diameter of 3 mm were cut from the initial cylindrical scaffolds. Before usage, cryogels were extensively washed with sterile PBS. For cell culture experiments, cryogel scaffolds were additionally incubated overnight in PBS supplemented with antibiotic‐antimycotic solution (1000 units mL^−1^ of penicillin, 1000 µg mL^−1^ of streptomycin, and 2.5 µg mL^−1^ of Amphotericin B from Gibco‐Life Technologies, USA), followed by washing in sterile PBS. Directly before cell seeding, the cryogels were equilibrated in the respective cell culture medium for at least 2 h in a cell culture incubator (37°C and 5% CO_2_).

### 4.3 Solution‐Based Mineralization of Cryogels

A 7 mm CaCl_2_ stock solution and a 7 mm K_2_HPO_4_ stock‐solution (Sigma–Aldrich Merck KGaA, Germany) were prepared by separately dissolving the reagents in a Tris(hydroxymethyl)aminomethane (Tris)‐buffered saline solution (50 mm Tris and 75 mm sodium chloride, Sigma–Aldrich Merck KGaA, Germany) and adjusting the pH to 7.4, similar to previously described [76]. For mineralization, PBS‐swollen and washed cryogel scaffolds were transferred onto cellulose filter paper (Whatman, United Kingdom) to remove PBS from the macropores, washed twice with CaCl_2_ stock solution and then added inside a clean 20 mL glass vial containing 10.296 mL CaCl_2_ stock solution stirred at 300 rpm. After pre‐incubation of the scaffolds in the CaCl_2_ stock solution for 30 min, 7.225 mL of the K_2_HPO_4_ stock solution was slowly added to start the mineralization reaction. The mineralization reaction was performed at room temperature in open air and the mineral solution was constantly stirred throughout the whole process. The scaffolds were removed after different incubation times and the mineralization reaction was stopped by short immersion in ice‐cold Tris saline‐buffered ethanol solution (1:1 ratio), followed by removal of this solution from the cryogel macropores using a cellulose filter paper and extensive washing in PBS. Scaffolds were stored in PBS at 4°C until usage.

### 4.4 Microstructure Analysis of Dry Cryogels via Scanning Electron Microscopy

For scanning electron microscopy (SEM), PBS‐swollen cryogel scaffolds were dehydrated via incubation in an ethanol dilution series (20%, 50%, 80% and 99%, 15 min incubation in each solution), followed by final drying overnight in a heating oven at 30°C (Vacutherm, Heraeus instruments, Germany). Dry scaffolds were coated with gold for 40 s at 40 mA (SCD 050 sputter coater, BAL‐TEC, Liechtenstein) and imaged in high vacuum mode with a secondary electron detector using a XL30 ESEM‐FEG microscope (Philips, Netherlands) or a MIRA3 microscope (TESCAN, Czech Republic).

### 4.5 X‐Ray Diffraction Measurements to Study Mineral Type and Crystal Size

PBS swollen cryogel scaffolds were cut at room temperature with a semiautomatic vibrating blade microtome (Vibratome VT1200, Leica Biosystems, Germany) to 400 µm sections. Afterward, sections were dried as described for SEM. WAXS measurements were performed using a laboratory X‐ray diffraction system (NanoStar, Bruker AXS, Germany) with a wavelength of 0.154 nm. WAXS patterns of all samples from the center and the boarder of the dry cryogel discs were recorded (total of n = 3 measuring points per sample). A correction of WAXS patterns for instrument‐related background scattering was performed and the integration of the 2D scattering data gave the intensity (*I*) of the signal as a function of the scattering angle (*2θ*) or of the scattering vector (*q*). To characterize the average mineral crystal length, WAXS analysis using a synchrotron radiation source (microfocus beamline µSpot, BESSY II, Germany) were performed and analyzed analogous to as described [77]. X‐ray diffraction patterns of dry cryogel sections were recorded using an Eiger X9M detector and a wavelength of 0.82656 Å, a beam size of 100 µm, an exposure time of 120 s and a sample‐to‐detector distance of 302.42 mm. For the analysis, the data reduction process of the software DPDAK (directly programmable data analysis kit) [78] was used to perform integration of the 2D WAXS patterns in azimuthal direction to obtain radial intensity profiles (I(q), with q being the scattering vector), as well as to carry out peak fitting (Gaussian function) of the (002)‐reflection to determine length of crystals (L‐parameter analysis). Diffraction patterns shown in the manuscript were normalized to the average intensity at the lowest 2*θ* or q‐values.

### 4.6 Quantification of Mineral Content

Mineralized cryogels were extensively washed and subsequently incubated in 0.1 m HCl solution at 37°C under constant shaking for 24 h to fully demineralize the scaffolds. As a control, a known amount of hydroxyapatite powder (Sigma–Aldrich Merck KGaA, Germany) was incubated. After the incubation, the supernatant was removed (including solution from macropores via centrifugation) and scaffolds were washed 2x with 0.1 m HCl solution. For quantification of calcium ions in the supernatants, a 0.4 mm Arsenazo III (MP Biomedicals, USA) solution dissolved in 0.02 m Tris‐buffer (pH 7.4) was prepared. To start the calcium assay, sample and Arsenazo III solution were mixed in a 1:3 ratio. A standard curve was prepared using a calcium chloride solution (Sigma–Aldrich Merck KGaA, Germany). Absorbance was read at 650 nm using a micro plate reader (Sunrise from TECAN, Switzerland).

### 4.7 Uniaxial Compression Testing of Cryogels

To evaluate the bulk mechanical properties of cryogels uniaxial compression tests were performed, according to as described using an ARES G2 Rheometer (TA Instruments, USA) [34]. The compressive elastic modulus was derived from the linear slope of the stress‐strain curves at low compression (strain between 0% and 10%). Measurements of cylindrical hydrogel scaffolds were performed analogous. To obtain 3D cylinders hydrogel solution was polymerized between two hydrophobic glass cover slips.

### 4.8 AFM‐Nanoindentation to Measure Elastic Moduli of Cryogels Struts and Hydrogels

Analysis of the local mechanical properties of PBS‐swollen thin cryogel sections and cylindrical hydrogel discs was performed utilizing a NanoWizard II AFM (JPK Instruments, Germany) mounted on an inverted light microscope (Axio Observer D.1, Zeiss, Germany) as described [34, 40].

### 4.9 SDF‐1 Release Experiments

Release experiments were performed according to previously established [34]. In here, non‐mineralized or 24 h mineralized macroporous starPEG‐sGAG cryogels were loaded with 450 ng SDF‐1. For the quantification of protein concentrations enzyme‐linked immunosorbent assays (ELISAs) were performed according to the manufacturer's instructions (Human SDF‐1 DuoSet ELISA, R&D Systems, USA).

### 4.10 RGD‐Functionalization of starPEG‐sGAG Cryogels

The functionalization of cryogels with RGD‐peptides (2 mol RGD per mol heparin, GWGGRGDSP, Mw: 886.92 g mol^−1^, synthesized in‐house) was performed prior to mineralization [34]. After the functionalization, the cryogels were excessively washed and stored at 4°C until further usage.

### 4.11 Assembly of Biphasic Hydrogel Constructs

For the assembly, poly(dimethylsiloxane) (PDMS, SYLGARD 184 silicone elastomer kit, Sigma–Aldrich Merck KGaA, Germany) based molds with a diameter of 4 mm and a height of 1 mm were placed onto hydrophobic glass coverslips and then filled with 20 µL of the starPEG‐sGAG bulk hydrogel precursor solution. Afterward the PBS‐swollen and optionally biofunctionalized and/or mineralized (24 h) cryogel scaffold (diameter ∼6 mm) was carefully added on top (Figure S2a). The hydrogel solution was allowed to fully polymerize before the PDMS ring was removed, and the biphasic cryogel/hydrogel constructs were transferred into cell culture medium.

### 4.12 Cell Culture

All cells used in this study were expanded and maintained inside a humidified cell culture incubator at 37°C and 5% CO_2_. Cell culture medium was fully replaced by fresh medium every other day. The cell line MDA‐MB‐231 was kindly obtained as a gift from the group of Prof. Joan Massagué. As reported, the cells express red fluorescent protein (RFP) due to the transfection with a retroviral vector [79]. MCF‐7 cells were obtained from the Deutsche Sammlung für Mikroorganismen und Zellkulturen (DSMZ; Braunschweig, Germany). MDA‐MB‐231 and MCF‐7 cells were cultured in growth medium (DMEM high glucose 4.5 g L^−1^ supplemented with 10% fetal bovine serum (FBS, Biochrom AG/Merck Millipore, Germany) and 1% penicillin–streptomycin (PS, Sigma–Aldrich Merck KGaA, Germany) and used within passage 20.

### 4.13 Cell‐Culture Experiments

Cell‐culture experiments were performed with MCF‐7 or MDA‐MB‐231 cells directly encapsulated in MMP‐degradable and RGD‐functionalized nanoporous bulk hydrogels using a hydrogel precursor solution that contained 3 × 10^4^ cancer cells. Depending on the type of experiment, 24 h mineralized and non‐mineralized fluorescently labeled cryogels were additionally functionalized with RGD or loaded with SDF‐1 as previously reported [34]. Biphasic cryogel/hydrogel constructs were prepared, as described and cultivated in growth medium, which was replaced every other day. After seven days, cells were washed with PBS to remove eventually loosely attached cells and fixed in 4% paraformaldehyde (PFA, Sigma–Aldrich Merck KGaA, Germany) solution for 20 min. The fixed samples were stored in PBS at 4°C until staining.

### 4.14 Fluorescence Staining

Fixed samples were blocked for 1 h in 10% goat serum solution (Dianova, Germany) diluted in DPBS and supplemented with 0.1% Triton‐X 100 (Sigma–Aldrich Merck KGaA, Germany). Subsequently, the samples were incubated with fluorescently labeled phalloidin, diluted 1:200 (Phalloidin ATTO‐488, ATTO‐550, or ATTO‐633 from ATTO‐TEC, Germany) in staining buffer (PBS supplemented with 1% goat serum solution). After 3 h, all the samples were washed three times in staining buffer and then the nuclei were stained for 40 min with Deep Red Anthraquinone 5 Fluorescent Probe (DRAQ5, Thermo Fischer Scientific, USA) diluted 1:1000 in staining buffer. Finally, all the samples were excessively washed and stored in PBS at 4°C until microscopic imaging.

### 4.15 Microscopy and Image Analysis

The fluorescently stained cryogel/hydrogel constructs were halved with a scalpel, washed with PBS and then the central region of each half – spanning the cross‐sectional area from the hydrogel (top) to the cryogel (bottom)—was imaged using a spinning disc confocal microscope (100 µm z‐stack, 2 × 3 tiles, 10x objective, Andor Dragonfly, Oxford Instruments, United Kingdom). The images were analyzed using the software Imaris (Bitplane, USA). Based on the signal of the fluorescently labeled cryogel, a region of interest data analysis was performed to separately detect stained nuclei in the hydrogel and the cryogel compartments using the spot analysis tool of the software. Based on the *x*‐ and *y*‐coordinates of each nuclei computed by the software, the spatial distribution of nuclei throughout the cryogel from top to bottom was quantified as a local frequency distribution (colonization profile, *CP*) using the software Prism 7.00, 8.00 or 10.6.1 (GraphPad, USA).

### 4.16 Quantification of Cell Proliferation

Proliferation of MDA‐MB‐231 cells was evaluated using the synthetic nucleoside bromodeoxyuridine (BrdU), which was supplemented to the MDA‐MB‐231 growth medium (1:100, Thermo‐Fischer Scientific, USA) and incubated for 15 h at 37°C and 5% CO_2_. The incorporated BrdU was detected using an immunofluorescent BrdU staining kit, which was used according to the manufacturer's instructions (eBioscience BrdU Kit for IHC/ICC Immunofluorescence eFluor 660, Thermo Fisher Scientific, USA). Following the antibody staining, the cells’ nuclei were stained via incubation in Hoechst 33 342 staining solution (diluted 1:1000 in PBS, Thermo Fisher Scientific, USA) for 40 min. Finally, all samples were washed three times and then stored in PBS at 4°C until imaging.

### 4.17 RNA‐Isolation

MDA‐MB‐231 cells were seeded into mineralized and non‐mineralized cryogels equilibrated in growth medium analogous to described in [80]. In brief, the medium was removed from the macropores of the scaffolds by transferring them onto sterile cellulose filter paper. A total of 1 × 10^5^ MDA‐MB‐231 cells were seeded into the semi‐dry cryogel transferred into a 24‐well suspension culture plate, by adding 12.5 µL of cell suspension on top, flipping the scaffold and adding another 12.5 µL on the other side to achieve better distribution of the cells throughout the cryogel. The cells were then let to adhere in a humidified chamber for 3 h at 37°C and 5% CO_2_. Afterward, the scaffolds were transferred into a new 24‐well plate and 1.5 mL of medium was added per well. Every other day, 1 mL per well of medium was removed and an equal volume of fresh medium was added. To ensure that the effects caused by the underlying cryogel matrices were not masked by effects caused by cell–cell interactions and/or cell‐secreted ECM and that at the same time a suffiently high number of cells can be harvested for analysis, culture was performed for 3 days. Then, the cryogels were washed with ice‐cold PBS, and the cells inside the cryogels were lysed by vortexing in lysis buffer (RLT buffer, RNeasy Micro Kit, Qiagen, Germany), following homogenization (QIAshredder, Qiagen, Germany). Subsequently, RNA isolation was performed following the manufacturer's instructions of the RNeasy Micro kit (Qiagen, Germany). Quantity and quality of isolated RNA was evaluated with an Agilent 2100 Bioanalyzer (RNA 6000 Nano Kit, Agilent Technologies, USA).

### 4.18 RNA‐Sequencing and Bioinformatic Analysis

For sequencing mRNA was isolated from on average 300 ng total RNA by poly‐dT enrichment using the NEBNext Poly(A) mRNA Magnetic Isolation Module (NEB) according to the manufacturer's instructions. Samples were then directly subjected to the workflow for strand‐specific RNA‐Seq library preparation (Ultra II Directional RNA Library Prep, NEB). For ligation NEB Next Adapter for Illumina of the NEB Next Multiplex Oligos for Illumina Kit were used. After ligation, adapters were depleted by an XP bead purification (Beckman Coulter) adding the beads solution in a ratio of 0.9:1 to the samples. Unique dual indexing was done during the following PCR enrichment (12 cycles) using amplification primers carrying the same sequence for i7 and i5 index (Primer 1: AAT GAT ACG GCG ACC ACC GAG ATC TAC AC NNNNNNNN ACA TCT TTC CCT ACA CGA CGC TCT TCC GAT CT, Primer 2: CAA GCA GAA GAC GGC ATA CGA GAT NNNNNNNN GTG ACT GGA GTT CAG ACG TGT GCT CTT CCG ATC T). After two more XP bead purifications (0.9:1), libraries were quantified using the Fragment Analyzer (Agilent). Libraries were sequenced on an Illumina NovaSeq 6000 in 100 bp paired‐end mode with an average of 50 million fragments per library.

FastQC (http://www.bioinformatics.babraham.ac.uk/) was used to run a basic quality control of the sequencing data. Fragments were aligned to the human reference (hg38) with support of the Ensembl 98 splice sites using the aligner gsnap (v2020‐12‐16) [81]. Fragments per gene and samples were obtained based on the overlap of the uniquely mapped fragments with the same Ensembl gene annotation using featureCounts (v2.0.1) [82] DESeq2 R package (v1.30.1) [83] and Independent Hypothesis Weighting (v1.18.0) [84, 85] were used for the following exploratory analysis and differential gene expression analysis. Here, the samples were divided into the three types: Non‐Mineral, Mineral and 2D control. For exploratory analysis, sample to sample Euclidean distance, Pearson and Spearman correlation coefficient (r) and PCA based upon the top 500 genes showing highest variance were computed to explore correlation between biological replicates and conditions. Genes with a maximum of 1% false discovery rate (padj ≤ 0.01) and fold change of < −1 and > 1 were considered as significantly differentially expressed for the comparisons Mineral vs Non‐Mineral and Non‐Mineral vs TCP. The dataset of differentially expressed genes (DEG) were further analyzed using the Ingenuity Pathway Analysis software (IPA, Qiagen, Germany) with the following analysis parameters: log 2‐fold change < – 1 or > 1, adjusted *p*‐value < 0.01 and baseMean > 10).

### 4.19 Statistical Analysis

Prism 7.00, 8.00 or 10.6.1 software (GraphPad, USA) was used to determine statistically significant differences. Unpaired *t* tests (Figures 1f and 2c–e), one‐way ANOVA (Figures 1e and 2b; Figure S1f,h,i,k) or two‐way ANOVA (Figures 2f–h and 3b–e; Figure S2d,e) analysis with Tukey's multiple comparisons test were performed. All data are presented as mean ± standard deviation, except for frequency distributions (mean ± standard error (SE)). A difference between two groups was considered as statistically significant when the *p*‐values were less than 0.05. Asterisks indicate statistical significance: * (*p* < 0.05), ** (*p* < 0.01), *** (*p* < 0.001) or **** (*p* < 0.0001) and *ns* stands for not statistically significant. In Figures 2f–h and 3b–e, Figures S1h,i,k and S2d,e not statistically significant differences were not explicitely indicated by “*ns*” in order to prevent overloading of the diagrams. In Figure 1e and Figure S1f we only show the statistically significant differences for 0 vs. 3 h, 3 vs. 24 h and 24 vs. 168 h.

## Author Contributions

Carsten Werner, Prof. (Conceptualization: Lead; Supervision: Lead; Writing – original draft: Equal; Writing – review and editing: Lead). Jana Sievers‐Liebschner, Dr. (Investigation: Lead; Validation: Lead; Writing – original draft; Lead; Writing – review and editing: Equal). Petra Welzel, Dr. (Investigation: Supporting; Methodology: Equal; Supervision: Supporting; Writing – original draft: Supporting; Writing – review and editing: Equal). Maximilian Fusenig, Dr. (Investigation: Supporting; Methodology: Supporting; Writing – review and editing: Equal). Linda Sturm (Investigation: Supporting). Dagmar Pette (Investigation: Supporting). Wolfgang Wagermaier, Dr. (Investigation: Supporting; Writing – original draft: Supporting). Claudia Fischbach, Prof. (Conceptualization: Equal; Methodology: Equal; Supervision: Equal; Writing – original draft: Supporting; Writing – review and editing: Supporting). Peter Fratzl, Prof. (Conceptualization: Equal; Supervision: Equal; Writing – original draft: Equal; Writing – review and editing: Equal)

## Conflicts of Interest

Carsten Werner is co‐inventor of a patent (WO2010060485A1) covering the hydrogel materials used in this study; he also holds shares in the spin‐off company ZetaScience GmbH, Dresden, offering customized hydrogel precursors.

## Supporting information




**Supporting file**: advs73978‐sup‐0001‐SuppMat.pdf.

## Data Availability

The data that support the findings of this study are available from the corresponding author upon reasonable request.
