# Supplementary material for: Mineralized Cryogel/Hydrogel Constructs to Recapitulate Early Breast Cancer Bone Metastasis In Vitro
Source: Adv Sci (Weinh). 2026 Jan 27;13(18):e19798. doi: 10.1002/advs.202519798 (PMC13042468; doi:10.1002/advs.202519798)
Supplement: Supplementary file 1 — Supporting file: advs73978‐sup‐0001‐SuppMat.pdf. [file ADVS-13-e19798-s001.pdf]

Supporting Information

Mineralized Cryogel/Hydrogel Constructs to Recapitulate Early Breast Cancer Bone Metastasis In Vitro

*Jana Sievers-Liebschner, Petra B. Welzel, Maximilian Fusenig, Linda Sturm, Dagmar Pette, Wolfgang Wagermaier, Claudia Fischbach, Peter Fratzl, Carsten Werner**

Supporting Results

Mechanical Properties of Mineralized starPEG-GAGs Cryogels

Uniaxial compression measurements revealed that all mineralized starPEG-sGAG cryogels were able to withstand high compressive forces (90% compression) without exhibiting damage (Figure S1g), as known from the non-mineralized cryogels.[1] The mineral layer was still intact after the compression testing implying a good integration of the mineral crystals with the cryogel matrix. Consistent, the overall bulk elastic moduli, derived from the analysis of the linear slope of the stress-strain curves at low compressions (<10%), were comparable for all cryogel samples (Figure S1h), even though a slight increase in the bulk elastic modulus was observed for 3h and 24 h mineralized cryogels that, however, was not statistically significant. Despite that no differences in the slope of the stress-strain curves at lower compressions were observed, a closer examination of the late stage of the compression measurement identified that at 88 % compression, at which the cryogel struts were in contact with each other, significantly higher stress values were found for cryogels mineralized for 3 h and 24 h, as compared to the non-mineralized condition (Figure S1i). As previously reported, the unique mechanical properties of the starPEG-sGAG cryogels result from the cryogelation-based fabrication technique leading to the formation of much denser polymer networks in the cryogel struts, as compared to those found in conventional bulk hydrogels formed at room temperature.[1] Together with the highly interconnected porous structure of the scaffolds, these characteristics give rise to the mechanically soft but very tough nature of cryogels. The compression analysis of the mineralized starPEG-sGAG cryogels hereby suggests that the tightly packed mineral platelets on the surface of the cryogel walls were able to intercalate during compression and thus did not act against it.

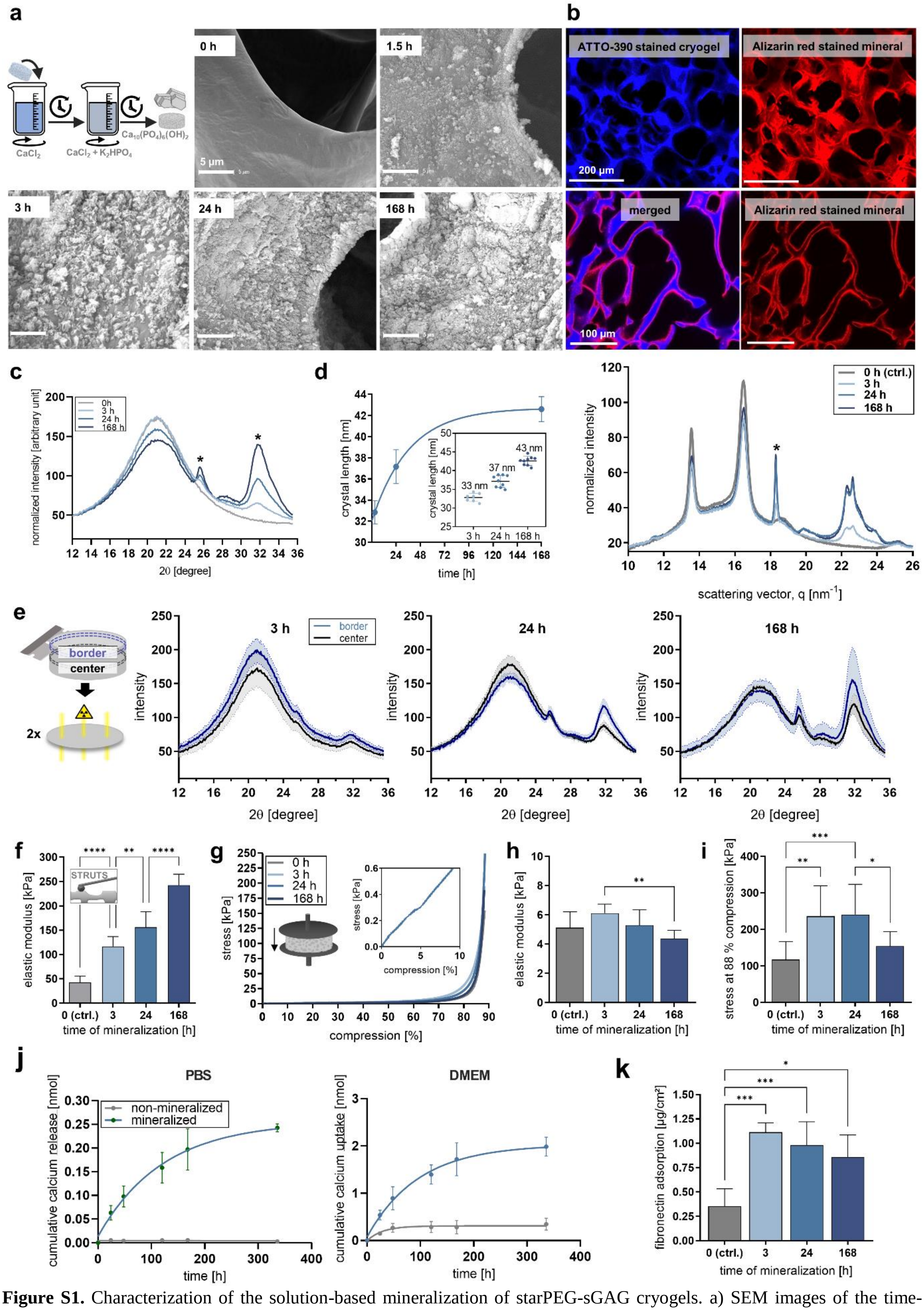

Figure S1. Characterization of the solution-based mineralization of starPEG-sGAG cryogels. a) SEM images of the time-dependent mineral crystal formation inside starPEG-sGAG cryogels, b) Confocal microscopy image of PBS-swollen 24 h-long mineralized cryogel stained with Alizarin Red solution. Top: Maximum intensity projection confocal microscopy image (100 µm z-stack) of fluorescently labeled cryogel (blue) and Alizarin Red stained calcium ions/mineral (red). Bottom: Cross-sectional confocal microscopy images of stained mineralized cryogel, c) Transmission WAXS diffraction patterns of non-mineralized and mineralized cryogels. Two principal diffraction peaks typical for hydroxyapatite appear at 2θ values of ~26°

and ~32° (asterisks), corresponding to (002)-crystal plane, d) Average crystal length of mineral crystals within starPEG-sGAG cryogels mineralized for different time periods (left) derived from the diffraction peaks at (002) reflection of synchrotron X-ray scattering analysis (right). Asterix: diffraction peaks at (002)-reflection, e) Analysis of mineral heterogeneity throughout one cryogel scaffold. Left: Schematic drawing illustrating the cutting of thin cryogel sections from the center and from the border of each sample. Subsequent, each section was analyzed via WAXS measurements at three different spots. Right: WAXS patterns of sections cut from mineralized cryogels (3 h, 24 h and 168 h) at the border or in the middle, demonstrating the presence of similar mineral crystals throughout one cryogel scaffold for each condition, f) Local mechanical properties of mineralized and non-mineralized cryogel struts as determined by means of AFM-nanoindentation measurements, g) Uniaxial compression measurements of the cryogels. Inset: Representative linear slope of the stress-strain curve at low compressions (<10%) from which the bulk elastic moduli of the cryogels (h) were derived, i) Stress at 88% compression, representing the point at which the cryogel struts are in contact with each other, j) Stability of 24-h long mineralized cryogels in PBS (without calcium) or calcium (1.8 mM) containing cell culture medium (DMEM, no serum), presented as cumulative release or uptake of calcium ions, k) Analysis of fibronectin adsorption to starPEG-sGAG cryogels mineralized for different time periods using the solution depletion technique. The adsorption is expressed in amount of protein adsorbed per unit of surface area.

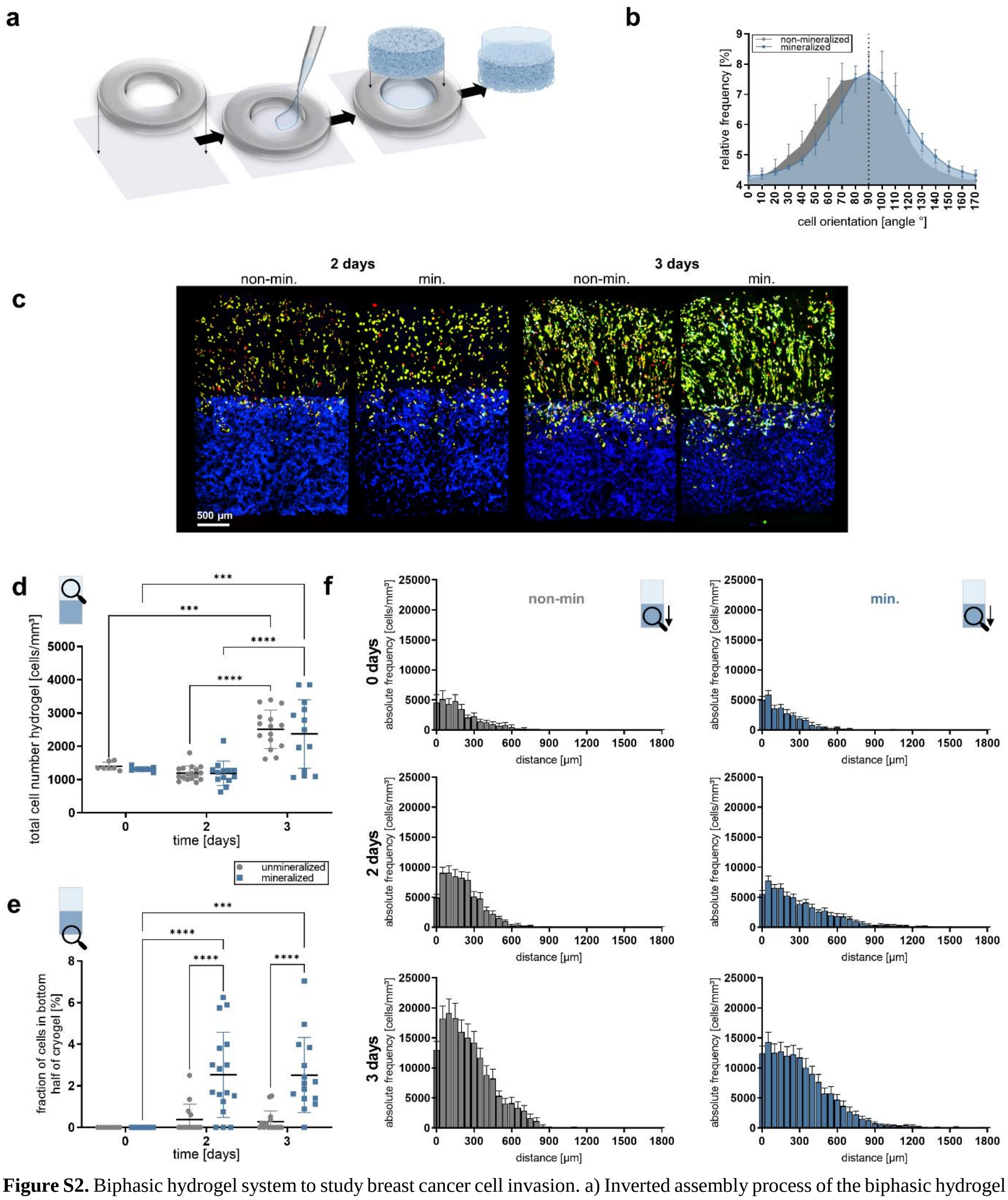

Figure S2. Biphasic hydrogel system to study breast cancer cell invasion. a) Inverted assembly process of the biphasic hydrogel scaffold utilizing a PDMS mold, b) Analysis of the alignment of MDA-MB-231 cells inside the bulk hydrogel compartment. Orientation angle frequency distribution of MDA-MB-231 cells inside the bottom 1000 µm of the bulk hydrogel compartment, c) Representative confocal microscopy image (100 µm maximum intensity projection) of the invasive response of MDA-MB-231 cells from the top hydrogels into non-mineralized or mineralized cryogels after two or three days in culture. Red: nuclei, green: F-actin and blue: cryogel, d-f) Quantification of the invasion response of MDA-MB-231 cells into non-mineralized and mineralized cryogels at 0, 2 or 3 days in culture: total cell number in hydrogel (d), fraction of cells in bottom half of cryogel (e) and colonization profiles (f).

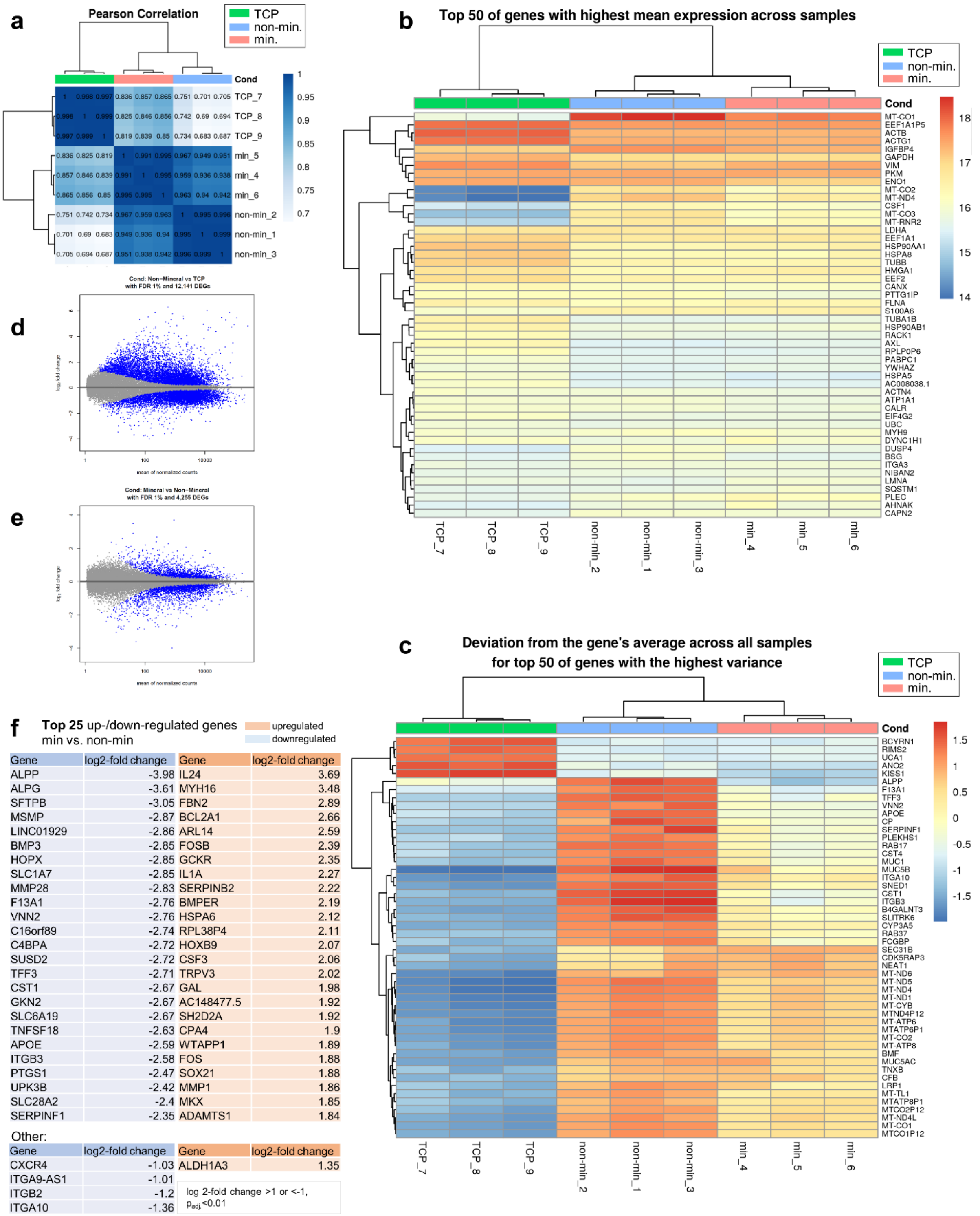

Gene	log2-fold change	Gene	log2-fold change
ALPP	-3.98	IL24	3.69
ALPG	-3.61	MYH16	3.48
SFTPB	-3.05	FBN2	2.89
MSMP	-2.87	BCL2A1	2.66
LINC01929	-2.86	ARL14	2.59
BMP3	-2.85	FOSB	2.39
HOPX	-2.85	GCKR	2.35
SLC1A7	-2.85	IL1A	2.27
MMP28	-2.83	SERPINB2	2.22
F13A1	-2.76	BMPER	2.19
VNN2	-2.76	HSPA6	2.12
C16orf89	-2.74	RPL38P4	2.11
C4BPA	-2.72	HOXB9	2.07
SUSD2	-2.72	CSF3	2.06
TFF3	-2.71	TRPV3	2.02
CST1	-2.67	GAL	1.98
GKN2	-2.67	AC148477.5	1.92
SLC6A19	-2.67	SH2D2A	1.92
TNFSF18	-2.63	CPA4	1.9
APOE	-2.59	WTAPP1	1.89
ITGB3	-2.58	FOS	1.88
PTGS1	-2.47	SOX21	1.88
UPK3B	-2.42	MMP1	1.86
SLC28A2	-2.4	MKX	1.85
SERPINF1	-2.35	ADAMTS1	1.84

Other:

Gene	log2-fold change	Gene	log2-fold change
CXCR4	-1.03	ALDH1A3	1.35
ITGA9-AS1	-1.01		
ITGB2	-1.2		
ITGA10	-1.36		

log 2-fold change >1 or <-1, p_{adj} <0.01

Figure S3. RNA sequencing analysis of the MDA-MB-231 cells phenotype on tissue culture plastic and mineralized and non-mineralized cryogels. Of each condition n=3 samples were sequenced, a) Heatmap of Pearson correlation showing the correlation coefficient of the gene expression patterns of mineralized, non-mineralized and TCP samples, b) Hierarchical clustered heat map of the top 50 genes with the highest mean expression across all samples. Color coding from blue to red with red representing the highest expression, c) Hierarchical clustered heat map of the top 50 most variable genes. The normalized fragment counts of the genes were transformed and the mean value across all samples was calculated. A value above 0 (yellow to red color) represents overexpression (relative to the average across all samples), where a negative value (white to blue) means the opposite, d) MA plot comparing the gene expression of TCP relative to non-mineralized samples, e) MA plot comparing the gene expression of mineralized compared relative to non-mineralized samples. The x-axis displays the mean expression and the y-axis the expression changes between conditions. Blue dots represent significantly DEGs based on a false discovery rate (FDR) of 1%, f) List of the Top 25 up/down-regulated genes when comparing mineralized to non-mineralized samples and considering exclusively a significance threshold of adjusted p < 0.01.

a Canonical pathway

Tumor microenvironment ↑

Gene	log2-fold change	Expected
CD274	↑ 1.410	↑ Up
CSF2	↑ 1.150	↑ Up
CSF3	↑ 2.060	↑ Up
FGF14	↓ -1.320	↑ Up
FOS	↑ 1.880	↑ Up
IDO1	↑ 1.000	↑ Up
IL1B	↑ 1.380	↑ Up
IL6R	↑ 1.220	↑ Up
ITGB3	↓ -2.580	↑ Up
LGALS9	↓ -1.260	↑ Up
MMP1	↑ 1.860	↑ Up
MMP3	↑ 1.730	↑ Up
MMP28	↓ -2.830	↑ Up
PDCD1LG2	↑ 1.570	↑ Up
PDGFB	↓ -1.490	↑ Up
PGF	↓ -1.840	↑ Up
PTGS2	↑ 1.210	↑ Up
RASD1	↓ -1.390	↑ Up
TNC	↑ 1.160	↑ Up

Acute phase response ↑

Gene	log2-fold change	Expected
C2	↓ -1.040	
C3	↓ -1.010	↑ Up
C1R	↓ -1.410	
C1S	↓ -1.100	
C4A/C4B+	↓ -2.090	
C4BPA	↓ -2.720	
C4BPB	↓ -1.170	
CP	↓ -2.230	↑ Up
FOS	↑ 1.880	↑ Up
IL1A	↑ 2.270	↑ Up
IL1B	↑ 1.380	↑ Up
IL6R	↑ 1.220	↑ Up
MAP2K6	↓ -1.100	↑ Up
RASD1	↓ -1.390	
SERPINE1	↑ 1.450	↑ Up
SERPINF1	↓ -2.350	
SERPINF2	↓ -1.220	

IL-17 signaling ↑

Gene	log2-fold change	Expected
CSF2	↑ 1.150	↑ Up
CSF3	↑ 2.060	↑ Up
CXCL3	↑ 1.620	↑ Up
FOS	↑ 1.880	↑ Up
IL11	↑ 1.140	↑ Up
IL1A	↑ 2.270	↑ Up
IL1B	↑ 1.380	↑ Up
LIF	↑ 1.180	↑ Up
LTB	↓ -1.730	↑ Up
MAP2K6	↓ -1.100	↑ Up
MMP3	↑ 1.730	↑ Up
MUC5B	↓ -1.710	↑ Up
PGF	↓ -1.840	↑ Up
PTGS2	↑ 1.210	↑ Up
RASD1	↓ -1.390	↑ Up
TNFSF10	↓ -1.120	↑ Up

Complement system ↓

Gene	log2-fold change	Expected
C2	↓ -1.040	↑ Up
C3	↓ -1.010	↑ Up
C1R	↓ -1.410	↑ Up
C1S	↓ -1.100	↑ Up
C4A/C4B+	↓ -2.090	↑ Up
C4BPA	↓ -2.720	↓ Down
C4BPB	↓ -1.170	↓ Down
CFH	↓ -1.610	↓ Down
ITB2	↓ -1.200	

LXR/RXR activation ↓

Gene	log2-fold change	Expected
ABCG1	↓ -1.580	↓ Down
APOC1	↓ -1.600	↑ Up
APOE	↓ -2.590	↑ Up
C3	↓ -1.010	↑ Up
C4A/C4B+	↓ -2.090	↑ Up
IL1A	↑ 2.270	↓ Down
IL1B	↑ 1.380	↓ Down
LYZ	↓ -1.430	↓ Down
PTGS2	↑ 1.210	
SCD	↓ -1.380	
SERPINF1	↓ -2.350	↑ Up
SERPINF2	↓ -1.220	↑ Up

Regulation of EMT by growth factors ↓

Gene	log2-fold change	Expected
CDH1	↓-2,020	↓ Down
EGR1	↑1,560	↑ Up
FGF14	↓-1,320	
FGFR3	↓-2,320	↑ Up
FOS	↑1,880	↑ Up
IL6R	↑1,220	↑ Up
LTB	↓ -1,730	↑ Up
MAP2K6	↓ -1,100	↑ Up
MMP1	↑1,860	↑ Up
PARD6B	↓-1,290	↑ Up
PDGFB	↓-1,490	↑ Up
RASD1	↓-1,390	↑ Up
TNFSF10	↓-1,120	↑ Up
TNFSF18	↓-2,630	↑ Up

b Disease and function

Cancer	Activation z-score
Growth of malignant tumor	↑ 2.157
Organismal death	↑ 4.458
Invasive tumor	↑ 2.040
Advanced malignant tumor	↑ 2.119
Metastasis	↑ 2.119

Cellular development	Activation z-score
Maturation of cells	-0.002
Differentiation of tumor cell lines	↑ 0.875
Differentiation of bone cells	↑ 1.808
Differentiation of osteoblasts	↑ 0.827
Differentiation of adipocytes	↓-1.224
Osteoclastogenesis	↑ 1.866

Cellular movement	Activation z-score
Migration of tumor cell lines	↑ 0.127
Cellular infiltration	↑ 1.976
Invasion of cells	↑ 0.414
Invasion of tumor cell lines	↑ 0.213
Invasion of breast cell lines	↑ 0.277
Chemotaxis	↓-1.538

Cell-to-cell signaling and interaction	Activation z-score
Cell-cell contact	↓-3.027
Assembly of intercellular junctions	↓-2.306
Development of gap junctions	↓-2.621
Adhesion of tumor cell lines	↓-0.891

Cellular Growth and Proliferation	Activation z-score
Cell proliferation of tumor cell lines	↓-0.743
Colony forming	↑ 0.652
Proliferation of progenitor cells	↑ 1.546
Expansion of cells	↑ 1.106
Proliferation of hematopoietic cells	↑ 2.146

Figure S4. Differential gene expression analysis of MDA-MB-231 cells cultivated in mineralized relative to non-mineralized cryogels. The analysis was performed utilizing QIAGEN's Ingenuity® Pathway Analysis (IPA®, QIAGEN Redwood City, www.qiagen.com/ingenuity) tool with considering exclusively a significance threshold of adjusted $p < 0.01$. Shown are the core canonical pathways (a) and associated diseased and cellular function (b) that were identified to be affected by presence of mineral crystals within the starPEG-sGAG cryogel. The z-scores represents the likelihood of the activation state of a given biological process.

Supporting Experimental Details

Characterization of mineral distribution via Alizarin Red Staining: Mineralized starPEG-sGAG cryogel scaffolds were incubated in an Alizarin Red staining solution (2 g per 100 ml in ultrapure water, pH 4.2, Sigma-Aldrich Merck KGaA, Germany) in the dark for 45 min under constant shaking. Afterwards, the scaffolds were washed four times with ultrapure water and finally stored in PBS at 4°C until imaging. Fluorescent microscopy images (100 µm z-stacks) of samples were taken using a spinning disc confocal microscope (10x Objective, Andor Dragonfly, Oxford Instruments, UK).

Analysis of mineral stability via quantification of calcium ions: To study the stability of the mineral formed inside starPEG-sGAG cryogels, the release or uptake of calcium ions into or from relevant solutions was quantified. Mineralized cryogels were incubated in PBS or Dulbecco's Modified Eagle's Medium (DMEM, Gibco-Life Technologies, USA) supplement with 1% penicillin-streptomycin (Sigma-Aldrich Merck KGaA, Germany) at 37°C and 5% CO2 inside a cell culture incubator for two weeks. After 1, 2, 5, 7, 9, 12 and 14 days the solutions were fully removed and new solution was added. The collected solutions were frozen and stored at -20°C until further analysis. To quantify calcium ions in the collected solutions, the Arsenazo III based assay was performed as described in the main method section.

Evaluation of fibronectin adsorption: Adsorption of the protein fibronectin to mineralized (24 h) and non-mineralized starPEG-sGAG cryogels was quantified using the solution-depletion technique. Fibronectin was purified from adult human plasma, according described[2], and fluorescently labeled with amine-reactive 5-(and-6)-carboxytetramethylrhodamine succinimidyl ester (TAMRA, Invitrogen AG, USA). For fibronectin adsorption experiments, mineralized and non-mineralized starPEG-sGAG cryogels were prepared and washed as described. After the removal of PBS from the macropores of the scaffolds via centrifugation for 3 min at 1500 rpm, the semi-dry scaffolds were transferred into fibronectin solution (0.23 µg $µl^{-1}$) at 37°C under slight shaking. After incubation for 24 h, the supernatant was fully removed via centrifugation of the scaffolds in tube filter inserts and then the scaffolds were washed two times with PBS. All solutions were collected, and the fibronectin concentration therein was quantified using a microplate reader (Spark multimode microplate reader, Tecan, Switzerland). For any detected decrease in fibronectin concentration, it was assumed that the depletion in the solution is the result of an adsorption to the cryogels. The fibronectin concentration of the

fibronectin solution incubated in parallel in empty reaction tubes were used as a reference (blank) and subtracted from all determined data.

Analysis of cell orientation in the hydrogel compartment of the biphasic hydrogel system: For analysis of cell alignment in the hydrogel compartment of the biphasic hydrogel constructs, confocal microscopy images (maximum intensity projections of 100 µm z-stacks) were analyzed using the software Fiji (ImageJ, NIH). For detection of cell shape the MDA-MB-231 cells inside the hydrogels were stained for F-actin. Due to a variance in the height of the hydrogel compartment and artefacts resulting from boundary effects to the cell culture medium, a region of interest analysis was performed starting at the interface to the cryogel up to a height of 1000 µm away from it (y-direction). For the image analysis, the directionality plugin of the software Fijii was used to automatically detect the orientation of the cells based on a Fourier spectrum analysis. The cell orientation was defined with respect to the orientation of the cryogel/hydrogel interface (0°), with 90°C referring to a cell that is oriented perpendicular to this interface.

Keller-Segel Model: The cryogel serves as a reservoir for the chemokine which diffuses into the hydrogel, while the hydrogel is a reservoir of cells that move into the cryogel driven by the cell concentration gradient and/or chemotaxis (that we describe by the Keller-Segel (KS) model[3]).

We call c the concentration of chemokine and ρ the density of cells. The model is one-dimensional across the boundary between the hydrogel and the cryogel, so that c and ρ both depend on spatial coordinate x (that we define such that $x = 0$ at the boundary) and on time t. The boundary conditions are such that $c = c_0$ deep inside the cryogel (corresponding to a saturation concentration based on the equilibrium with the polymer that contains the chemokine) and $c = 0$ deep inside the hydrogel. Conversely, $\rho = \rho_0$ deep inside the hydrogel (the initial loading with cells) and $\rho = 0$ deep inside the cryogel. Moreover, we have different diffusivities of cells within hydrogel and cryogel (that also depend on whether the cryogel is mineralized or not), D_h and D_c. To simplify, we take the same diffusion constant for the chemokine in both gel compartments, D_s. The concentration profiles are schematically shown in **Figure S5**.

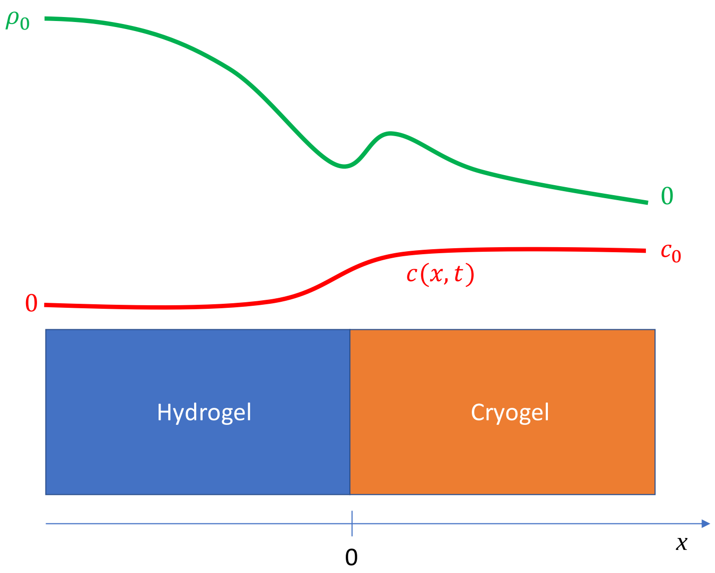

Figure S5. Schematic representation of the concentration profiles of chemokine (red) and the cancer cells (green) in the two gel compartments.

The KS model writes:

$$\frac{\partial \rho}{\partial t} = \nabla\,(D_i\,\nabla\rho - \chi\rho\,\nabla c) + f \quad (\text{where } i = h \text{ or } c, \text{depending on } x)$$

$$\frac{\partial c}{\partial t} = \nabla\,(D_s\,\nabla c) + g$$

The parameters f and g describe cell proliferation and chemokine secretion, respectively. At this point, we take $f = 0$, although we cannot exclude some level of proliferation that could be taken into account in future work. The parameter χ is the chemotactic sensitivity (which, in principle, could also be different depending on the presence/absence of mineral).

For our one-dimensional model, this reduces to:

$$(1) \quad \frac{\partial \rho}{\partial t} = \frac{\partial}{\partial x}\left(D_i\,\frac{\partial \rho}{\partial x} - \chi\rho\,\frac{\partial c}{\partial x}\right) \quad (\text{where } i = h \text{ or } c, \text{depending on } x)$$

$$(2) \quad \frac{\partial c}{\partial t} = D_s\,\frac{\partial^2 c}{\partial x^2}$$

Equation (2) can be solved immediately with the given boundary conditions (that is, $c = 0$ deep inside the hydrogel and $c = c_0$ deep inside the cryogel:

$$c(x,t) = \frac{c_0}{2}\left(1 + \text{erf}\left[\frac{x}{\sqrt{4\,D_s\,t}}\right]\right),$$

where

$$\mathrm{erf}\,[z] = \frac{2}{\sqrt{\pi}} \int_0^z e^{-t^2}\, dt$$

is the error function. We simplify notations by using $\tau = 4\, D_s\, t$, so that

$$\frac{\partial c}{\partial x} \approx \frac{c_0}{\sqrt{\pi\, \tau}} e^{-x^2/\tau}$$

and by inserting this into Eq. (1) above

$$(3) \quad \frac{\partial \rho}{\partial \tau} = -\frac{\partial J}{\partial x}, \text{ with } J = -\frac{D_i}{4D_s}\frac{\partial \rho}{\partial x} + \frac{\chi\, c_0\, \rho}{4D_s\, \sqrt{\pi\, \tau}}\, e^{-x^2/\tau} = -\lambda\, \frac{\partial \rho}{\partial x} + \frac{\mu\, \rho}{\sqrt{\tau}}\, e^{-x^2/\tau}\ .$$

The first term is the diffusion-like movement of the cells and the second term is the cell movement in a chemokine gradient (that depends on time). The (dimensionless) constants λ and μ are just abbreviations.

References

1 Sievers, J. *et al.* Customizing biohybrid cryogels to serve as ready-to-use delivery systems of signaling proteins. *Biomaterials* **278**, 121170-121170, doi:10.1016/j.biomaterials.2021.121170 (2021).

2 Brew, S. A. & Ingram, K. C. Purification of human plasma fibronectin. *Journal of Tissue Culture Methods* **16**, 197-199, doi:10.1007/BF01540650 (1994).

3 Keller, E. F. & Segel, L. A. Model for chemotaxis. *Journal of Theoretical Biology* **30**, 225-234, doi:10.1016/0022-5193(71)90050-6 (1971).
